# Population-based temporal trends and ethnic disparity in breast cancer mortality in South Africa (1999-2018): Joinpoint and age–period–cohort regression analyses

**DOI:** 10.3389/fonc.2023.1056609

**Published:** 2023-02-03

**Authors:** Gbenga Olorunfemi, Elena Libhaber, Oliver Chukwujekwu Ezechi, Eustasius Musenge

**Affiliations:** ^1^ Division of Epidemiology and Biostatistics, School of Public Health, University of Witwatersrand, Johannesburg, South Africa; ^2^ Faculty of Health Sciences, University of Witwatersrand, Johannesburg, South Africa; ^3^ Division of Clinical Sciences, Nigerian Institute for Medical Research, Lagos, Nigeria

**Keywords:** APC analysis, age period cohort analysis, breast cancer mortality rate, ethnic disparity of cancer, female cancer trends, gynecological cancer trends, join point regression, South Africa

## Abstract

Globally, breast cancer is the leading cause of cancer deaths, accounting for 15.5% of female cancer deaths in 2020. Breast cancer is also the leading cause of female cancers in South Africa. The rapid epidemiological transition in South Africa may have an impact on the trends in breast cancer mortality in the country. We therefore evaluated the trends in the breast cancer mortality in SA over 20 years (1999–2020).

**Methods:**

Joinpoint regression analyses of the trends in crude and age-standardized mortality rates (ASMR) of breast cancer among South African women were conducted from 1999 to 2018 using mortality data from Statistics South Africa. Age–period–cohort regression analysis was then conducted to evaluate the independent effect of age, period, and cohort on breast cancer mortality, and analysis was stratified by ethnicity.

**Results:**

The mortality rate of breast cancer (from 9.82 to 13.27 per 100,000 women) increased at around 1.4% per annum (Average Annual Percent Change (AAPC): 1.4%, 95% CI:0.8–2.0, P-value< 0.001). Young women aged 30–49 years (1.1%–1.8%, P-value< 0.001) had increased breast cancer mortality. The risk of breast cancer mortality increased among successive birth cohorts from 1924 to 1928 but decreased among recent cohorts born from 1989 to 1993. In 2018, the breast cancer mortality rate among Blacks (9.49/100,000 women) was around half of the rates among the non-Blacks. (Coloreds: 18.11 per 100,000 women; Whites: 17.77/100,000 women; Indian/Asian: 13.24 per 100,000 women).

**Conclusions:**

Contrary to the trends in high- and middle-income countries, breast cancer mortality increased in South Africa especially among young women. Breast cancer prevention programs should be intensified and should also target young women. The marked disparity in ethnic burden of breast cancer should be considered during planning and implementation of interventions.

## Introduction

Globally, breast cancer is the leading cause of cancer deaths, accounting for 15.5% of female cancer deaths in 2020 (1). In high-income countries (HIC), the age-standardized incidence rates of breast cancer are very high as compared with the rates in low- and middle-income countries (LMICs) (1). Nonetheless, the 5-year survival rate of breast cancer exceeds 90% in HICs but is much lower in LMICs (30%–60%) (1). The noted disparity is related to variation in the prevalence of etiological factors, cancer screening facilities, stage at presentation, and access to treatment (1, 2). In South Africa, breast cancer had the second highest national ASMR of 16.0 per 100,000 women after cervical cancer (1).

The etiology of breast cancer is a complex interaction between hormonal, reproductive, and environmental factors (1, 3, 4). Additionally, smoking, prolonged use of oral contraceptive pills, and ethnicity have been associated with breast cancer burden (1, 3–5). Furthermore, genetic predisposition, positive family history, chronic exposure to estrogen such as delayed age at first pregnancy, early menarche, low parity, and short-term breastfeeding practices have been implicated in the evolution of breast cancer (1, 3–6).

South Africa is a middle-income multiethnic country (7, 8). Since the commencement of multiethnic democracy in 1994, the successive South African government promoted policies aimed at reducing socioeconomic inequality and increased access to sexual and reproductive health (SRH) services among the various ethnic groups (9, 10). Hence, current evidence of ethnic disparity in the trends and burden of breast cancer in South Africa can be useful in developing targeted interventions (11). The current epidemiological and health transition in South Africa has led to increased prevalence of obesity, westernization of diet, sedentary lifestyle, and changes in reproductive behavior (such as reduced parity and increased use of COCPs) (12). South Africa has one of the highest prevalences of human immunodeficiency virus (HIV) globally. Although HIV is not implicated in the evolution of breast cancer, studies reported worse survival among HIV-positive women (2).

Cancer surveillance and trend analyses are useful for providing evidence to aid immediate and long-term cancer control efforts (7, 13–16). A useful statistical tool for objectively evaluating cancer trends to inform policy is joinpoint regression modeling. Joinpoint regression modeling can assist to quantify statistically significant segmental and overall trends. Age–period–cohort (A–P–C) modeling is another very useful trend analytic tool for disentangling the impact of age, period, and birth cohorts on cancer trends. It is believed that age (“age effect”) can have a biological impact on the risks of many diseases. Thus, evaluating age-specific risks of cancers is important. Furthermore, “period effect” is the impact of public health interventions or population-level policies that can affect the overall risk of diseases among all age groups over a period of time. Some public health initiatives in South Africa such as expansion and easy access to oncological services, commencement of large-scale rollout of free anti-retroviral treatment (ART) in 2004, the implementation of the World Health Organization Framework Convention on Tobacco control from 2005, and the initiation of national breast cancer control policies can cause a "period effect" in the temporal trends in breast cancer mortality in South Africa. A group of people who were born around the same time tend to have a similar exposure to common biological, social, and economic events and may also have a similar reproductive behavior. Such “cohort effect” may lead to unique or cohort-specific risks of a disease. However, majority of national studies on the mortality trends of breast cancer in South Africa and other Sub-Saharan African (SSA) countries did not utilize the joinpoint and A–P–C modeling techniques (11). South Africa is one of the only three countries in Sub-Saharan Africa with a comprehensive civil registration and vital statistics system (CRVS) that can be utilized for research to improve the health needs of the people (17). We therefore aimed to evaluate the trends in breast cancer mortality in South Africa and stratified by ethnicity over a 20-year period (1999–2018) by utilizing both joinpoint and A–P–C regression modeling techniques.

## Materials and methods

This study is a temporal trend analysis of breast cancer deaths in South Africa from 1999 to 2018. South Africa is a multiracial middle-income country with an estimated population of 57.79 million in 2018, and around 51% were women (8). In 2018, the proportion of women by population group was as follows: Blacks (Black Africans) (80.4%), Coloreds (mixed ancestry) (8.9%), Whites (European descent) (8.3%), and Indians/Asians (2.4%) (18).

### Data source

Data on breast cancer mortality were obtained from the vital statistics records as collected and published by Statistics South Africa (Stats SA). By law, it is mandatory for all deaths in South Africa to be reported to the Department of Home Affairs (DHA) (18). Causes of death were coded by experienced staff of Stats SA using the International Classification of Diseases, Tenth Revision (ICD-10) (19). The code for the underlying cause of death for female breast was ICD10, C50 (11, 20, 21).

The population denominators for calculating the mortality rates were the mid-year population estimates of women (≥15 years) stratified by ethnicity and 5-year age group, as obtained from published data of Stats SA from 2002 to 2018. The annual mid-year population estimates for 1999–2001 were obtained by assuming the constant inter-census rate and increment between two South African population census of 1996 and 2001.

### Data quality

The vital registration methodology and records of South Africa have been internationally adjudged to be comprehensive (17). Joubert et al. found that civil registration of South Africa is satisfactory in terms of coverage, completeness of death registration, temporal consistency, age/sex classification, timeliness, and subnational availability (20). The Stats SA data are at present the only source of nationally representative cancer mortality records in South Africa.

#### Ethical considerations

Ethical approval for the conduct of this study was obtained from the Human Research and Ethics Committee (Medical) of the University of the Witwatersrand (clearance certificate number: M190544). Anonymized data with no risk of re-anonymization were utilized.

### Statistical analysis

Data were imported into Stata version 16 (StataCorp, USA) statistical software for statistical analysis. Data validation and data cleaning were done. Descriptive statistics such as frequency and mean (± standard deviation) were analyzed. The annual proportion of breast cancer in relation to all women and gynecological cancer mortality was calculated.

### Annual crude and age-standardized rates

The annual crude mortality rate (CMR) was calculated by dividing the annual breast cancer mortality among women aged ≥15 years by the mid-year female population (≥15years). The calculation was stratified by ethnicity (Whites, Coloreds, Blacks, and Asian/Indians) from 1999 to 2018.

Age-specific mortality rate was also calculated by dividing the cumulative age-stratified mortality of each 5-year age group (15–19, 20–24, 25–29…….75+) by cumulative age-stratified mid-year population of each age category. The annual age-standardized mortality rates (ASMR) were calculated using the direct method of standardization, and the Segi world standard population was the weighted population.


$$\text{Thus, the age-standardized rates}=\frac{\sum_{i=1}^{A}a_{i}w_{i}}{\sum_{i=1}^{A}w_{i}}\;\times 100,000$$

where *a_i_* is the age-specific rate of the *i* th 5-year age group and *w_i_* is the corresponding number of persons (or the weight) in the same 5-year age group *i* of the Segi world standard population.

The standardized rates were stratified by ethnicity. All rates were expressed per 100,000 women.

### Joinpoint regression modeling

A joinpoint regression analysis of the trends in the overall cancer mortality of breast cancer (stratified by ethnicity and age groups) was performed with the Joinpoint Regression software, version 4.9.0.1 (Statistical Methodology and Applications Branch, Surveillance Research Program, National Cancer Institute, Bethesda, MD). The Joinpoint Regression software fit a Poisson regression in which ln(rate) is the outcome and the year of occurrence is the explanatory variable. Log-linear modeling with four maximum joinpoints and 4,499 Monte Carlo permutation tests were conducted for each of the trends in ASMR.

The equation of the joinpoint trend is: (22–24)


(i) ln(Rate)=β×(Calendar year)+C

where β = coefficient of the calendar year, ln = natural logarithm, and C = constant (or intercept)

The annual percent change (APC) of the cancer rates between a previous calendar year “X” and the next calendar year “X+1” is


(ii) $$=\;\left({\text{Rate}}_{\left(x+1\right)}-{\text{ Rate}}_{\left(x\right)}/{\text{Rate}}_{\left(x\right)}\right)*100$$

From Eq. (i),


$${\text{Rate}}_{\left(x+1\right)}=e^{\beta \left(\times +1\right)\;+\;c}{\text{and Rate}}_{\left(X\right)}=e^{\beta \left(\times \right)\;+\;c}$$

$$\text{Hence, APC }=\;((e^{\beta \left(\times +1\right)\;+\;c}-\;e^{\beta \left(\times \right)\;+\;c})/e^{\beta \left(\times \right)\;+\;c})\;\times \;100=\;(e^{\beta \;–}1)\;\times \;100$$

where e = 2.7

The APC is equivalent to the Average Annual Percent Change (AAPC) if there are no joinpoints. However, when there are joinpoints, the segmental APC (with 95% confidence interval, CI) was calculated and the AAPC was iteratively calculated as a weighted average of all the segmental APCs. Conventionally, a positive or negative AAPC (or APC) with P-value<0.05 was taken as a statistically significant increased or decreased trend. If the P-value of the AAPC was >0.05, the trend is taken as a non-significant increased or decreased trend. If the AAPC is between -0.5 and + 0.5 with P-value >0.05, the trend is reported as stable.

### Age period cohort modeling of breast cancers

A–P–C modeling has been used by social scientists, demographers, and epidemiologists to assist in understanding the impacts of age, period, and birth cohort on the trends in prevalence of social, demographic, or disease outcome or rates. This modeling technique assists to disentangle the effect of chronological age (age effect), from “period effect” (impact of improvement in public health interventions, screening, diagnostic tools, and treatment modalities over time) and “cohort effect” (influence of socio-behavioral and reproductive characteristics and environmental impact on the health outcomes of a cohort of people that were born at the same time) (25–29).

Arithmetically, the relationship between age, period, and birth cohort is given as

Age = period (year of event) – cohort (year of birth). (or birth cohort = period – age) …(iii)

However, the A–P–C model assumes a Poisson distribution of the mortality rates (dependent variable) with age, period, and birth cohort as the covariates/independent variables.

The general equation of the A–P–C model is expressed as


(iv) $$Y=\;\alpha_{0}+\alpha X_{1}+\beta X_{2}+\gamma X_{3}+\epsilon .$$

where *Y* is the breast cancer mortality; X_1_, X_2_, and X_3_ are the age period and birth cohort with their corresponding effect estimates of *α, β*, and *γ*, respectively; *α*_0_ is the intercept; and *ε* is the residual.

Indeed, the natural logarithm of the mortality rates in Eq. (iv) becomes a linear or additive function of age, period, and birth cohort as expressed thus:


(v) $$\ln\left[E\;\left(M_{ij}\right)\right]=\text{ln}(D_{ij}/P_{ij})=\;\mu +\alpha_{i}+\beta_{i}+\;\gamma_{k}$$

where

*E*[*Mij*] represents the expected mortality rate at 5-year age group *i* (15–19, 20–24, 25–29….75+ years) and period *j* (1999–2003, 2004–2008, 2009–2013, 2014–2018); *D_ij_* and *P_ij_* are the number of deaths and corresponding population size in the *i* age group and the *j* period, respectively.*α_i_* represents the age effect in the age group *I*; *β_j_* denotes the period effect during a *j* period, *γ_k_* corresponds to the cohort effect among the *k_th_* (*k* =*i*+*j*–1) birth cohort, and *μ* is the intercept.

### Identifiability problem

The above arithmetic equation (iii) of the relationship between age, period, and cohort shows a perfectly linear relationship or linear dependency with inherent problem of collinearity when all of age, period, and birth cohort are added as covariates during the Poisson regression modeling of mortality rates. Therefore, reliable unique estimates for each of age, period, or cohort will ordinarily be difficult to obtain from the regression model because the covariate matrix will not be full-rank. This phenomenon is known as “identification” problem. Multiple methods have been proposed to circumvent the identification problems, but each has its merits and demerits (25). The methods proposed for “overcoming” the “identification” problems broadly entails the application of constraints on one of period, cohort, or both, and utility of estimable function techniques (25). Holford proposed and validated the estimable function algorithm by proving that if age, period, and cohort trends are orthogonally decomposed into linear and non-linear parts, many useful functions and estimates will be produced. To address the identification problems, we utilized the age–period–cohort webtool (Biostatistics Branch, National Cancer Institute, Bethesda, MD, USA). (Age Period Cohort Analysis Tool (cancer.gov) with accompanying R Studio codes (https://github.com/CBIIT/nci-webtools-dceg-age-period-cohort) as developed by Rosenberg et al. to produce estimable parameters of the A–P–C of the trends in breast cancer mortality. The webtool utilized the weighted least squares estimator (26).

Before the A–P–C analysis, data of breast cancer mortality were further prepared. Age was categorized into 5-year age groups from 15 to 75 years (15–19, 20–24, 25–29, 30–34 years, 35–39, 40–45,……75 years and above), and the year of mortality (calendar period) was also categorized into 5-year categories from 1999 to 2018 (1999–2003, 2004–2008, 2009–2013, 2014–2018). A Lexis matrix was then formed with age category of the mortality data as columns and the corresponding period as rows (the diagonal represents the corresponding birth cohort). The corresponding population at risk was also calculated for each age group and period. The above A–P–C data preparation was done for each of breast cancers and then stratified by ethnic groups (Blacks, Whites, Coloreds, and Indian/Asian). These data were then imputed in turn into the A–P–C webtool (26).

Several estimates are obtainable from the A–P–C regression modeling webtool (26). However, we reported the following: (1) net drift, which is equivalent to the overall log-linear trends after adjusting for period and cohort effect—net drift is also equivalent to the AAPC of the mortality trend; (2) local drift, which is equivalent to the APC of mortality trends in each age group; (3) longitudinal age-specific rates (longitudinal age-specific rates in the reference cohort, adjusted for period deviations, i.e., age trend + period trend); (4) cross-sectional age-specific rates (age trend − period trend); (5) cohort effect rate ratio; and (6) period effect rate ratio. The default (middle value) references for the period and cohort estimates were 2004–2008 and 1959–1963, respectively. Wald’s test of statistical significance, including the 95% CI of all the estimates, was also reported. Conventionally, the dominant patterns of the trends in the estimable parameters are descriptively reported. Afterward, the pattern is confirmed or further described based on the P-value and 95% CI of the estimates. A two-tailed test of significance was assumed, and P-value< 0.05 was taken as a statistically significant level. Analysis was conducted in both Stata (StataCorp, TX, USA) version 16 and R version 3.6.3 (R Foundation, Vienna, Austria).

## Results

During the 20-year period from 1999 to 2018, 4,386,517 deaths were reported among South African women who were 15 years and older. Of these female mortalities, around 8.24% (95% CI: 8.21%–8.26%, n = 361,449) were cancer-related mortalities. Deaths due to breast and gynecological cancers constituted around 37.39% (95% CI: 36.83%–38.13%, n = 134,778) of the cancer mortalities among women in the country. Breast cancer (n = 58,628, 41.27%, 95% CI: 41.01%–41.54%) was responsible for around 41.27% of breast and gynecological deaths. The proportion of breast and gynecological cancer deaths due to breast cancer appears to be stable during the study period (**Table 1**).

**Table 1:** Trends in the mortality rates and mean age at death of breast cancer in South Africa (1999–2018).

Year	Breast (n = 55,628)			
	Mortality (% of gyne and breast) ≥15 years	Age (mean ± SD)	CMR (per 100,000 women)	ASMR (per 100,000 women)
1999	1,848 (40.03)	59.43 **±** 15.55	12.02	9.82
2000	1,897 (39.89)	59.08 **±** 16.22	12.02	9.81
2001	2,131 (41.59)	59.61 **±** 15.53	13.16	10.89
2002	2,062 (39.63)	59.83 **±** 15.25	12.56	10.41
2003	2,113 (39.41)	59.66 **±** 15.78	12.43	10.14
2004	2,501 (42.56)	58.99 **±** 15.38	15.07	13.64
2005	2,551 (42.26)	59.46 **±** 15.41	15.7	12.89
2006	2,474 (40.68)	58.77 **±** 15.43	15.02	12.26
2007	2,707 (43.67)	59.93 **±** 15.44	16.21	13.14
2008	2,664 (42.61)	59.78 **±** 15.44	15.23	12.58
2009	2,729 (40.74)	59.56 **±** 15.48	15.37	12.59
2010	2,923 (43.14)	59.55 **±** 15.21	16.27	13.46
2011	2,997 (42.24)	60.33 **±** 15.24	16.44	13.16
2012	3,026 (42.12)	60.16 **±** 15.23	16.4	13.13
2013	3,168 (41.95)	60.22 **±** 15.34	16.28	12.58
2014	3,344 (40.83)	60.28 **±** 15.55	17.04	12.74
2015	3,424 (40.63)	60.26 **±** 15.47	17.27	13.25
2016	3,669 (41.22)	60.41 **±** 15.35	18.15	13.98
2017	3,610 (39.73)	60.18 **±** 15.69	17.56	13.29
2018	3,790 (40.48)	59.98 **±** 15.67	18.02	13.27

CMR, crude mortality rate; ASMR, age-standardized mortality rate.

### Trends in breast cancer mortality in South Africa, 1999–2018

**Figure 1:**
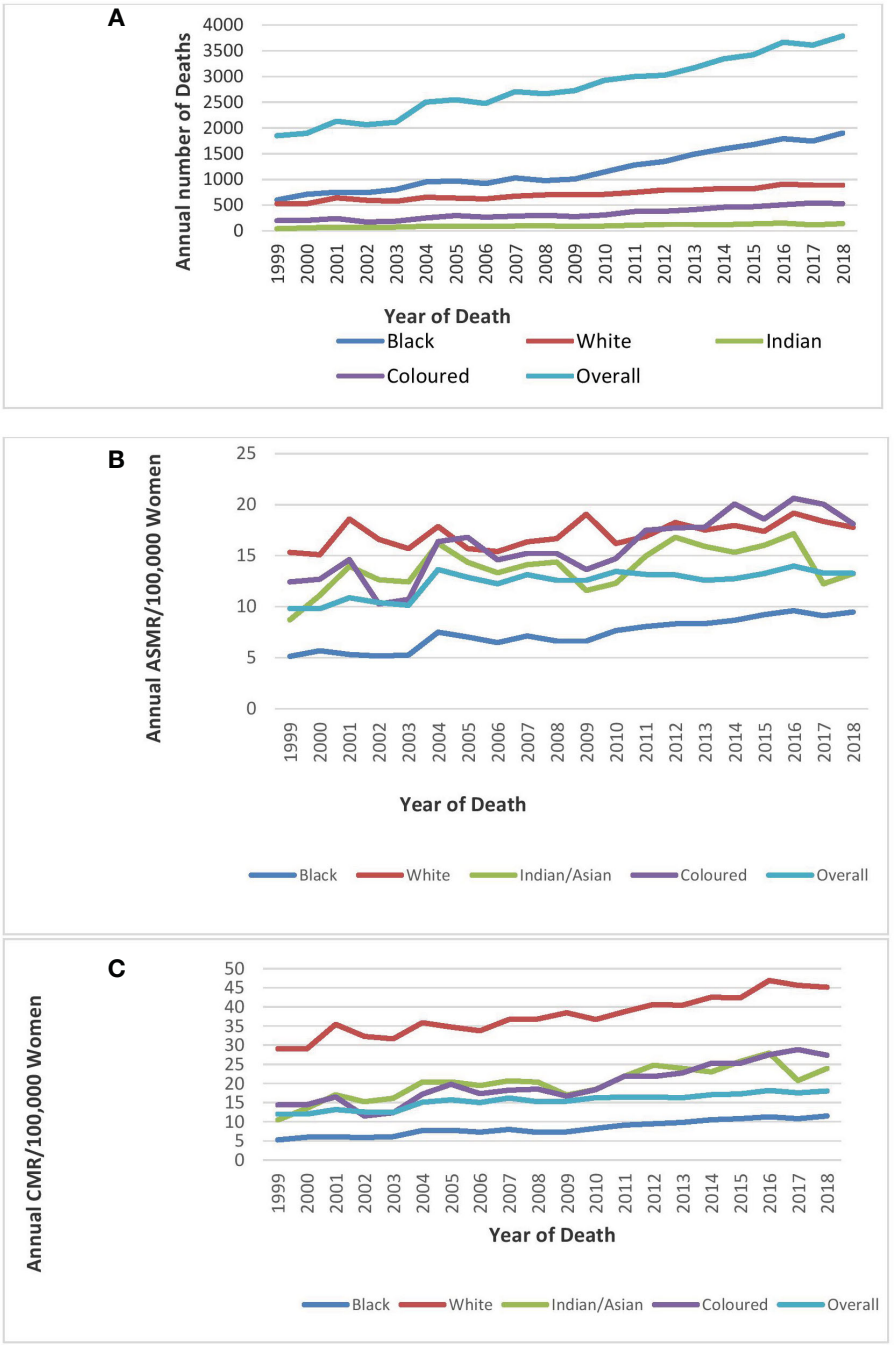
Trends in national and ethnic annual deaths **(A)**, age-standardized rates **(B)**, and crude mortality rates **(C)**of breast cancer in South Africa (1999–2018).

Mortality from breast cancer increased from 1,848 in 1999 to 3,790 in 2018 (**Figure 1A**; **Table 1**).

**Figure 2:**
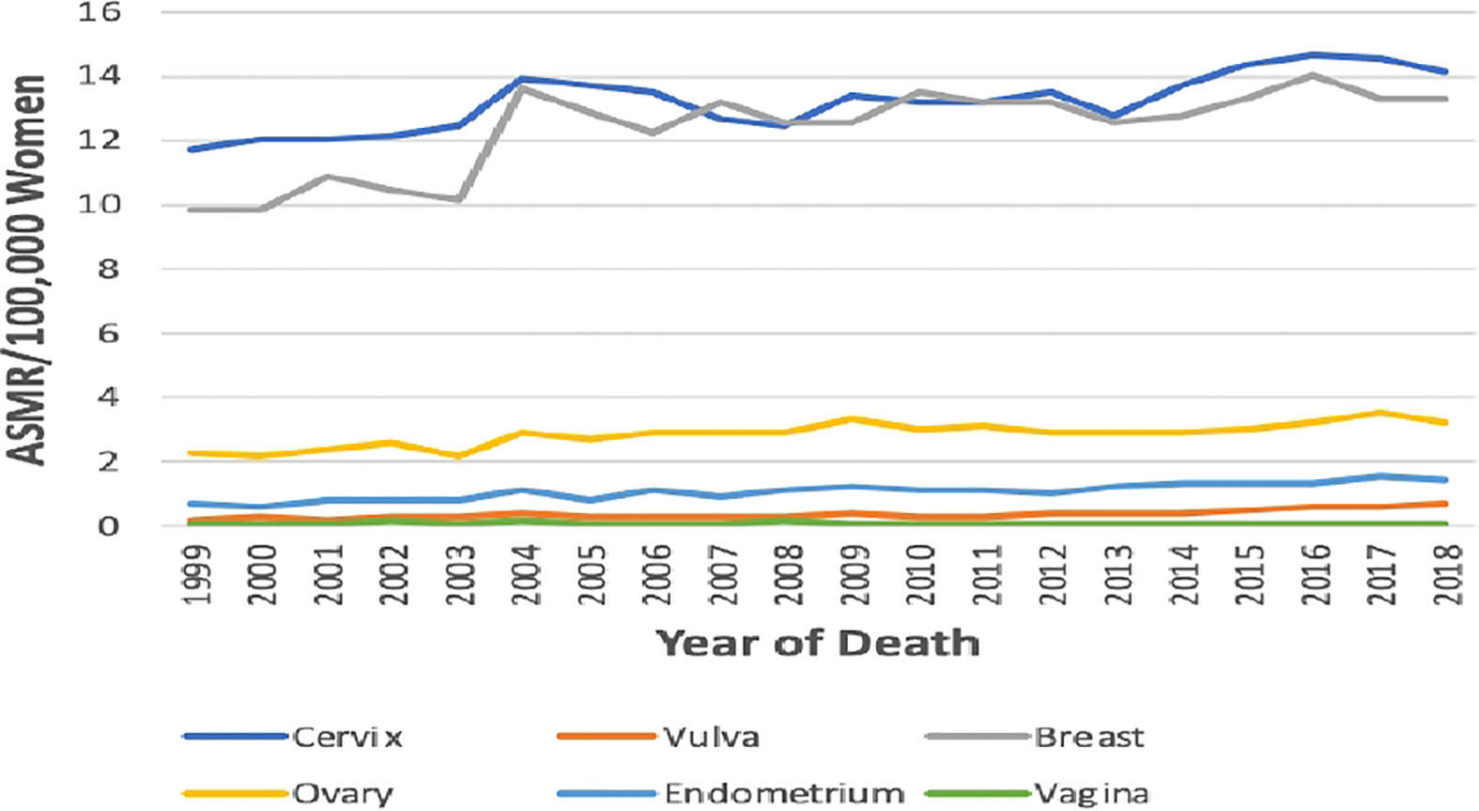
Trends in age-standardized mortality rates of breast and Gynaecological cancers in South Africa from 1999 to 2018.

The ASMR of breast cancer was second highest behind cervical cancer (**Figure 2**), and it increased from 9.8 deaths per 100,000 women in 1999 to 13.3 deaths per 100,000 women in 2018 at an average increase of 1.4% per annum (AAPC: 1.4%, 95% CI:0.8–2.0, P-value< 0.001) (**Table 1**; **Figure 1**).

**Figure 3:**
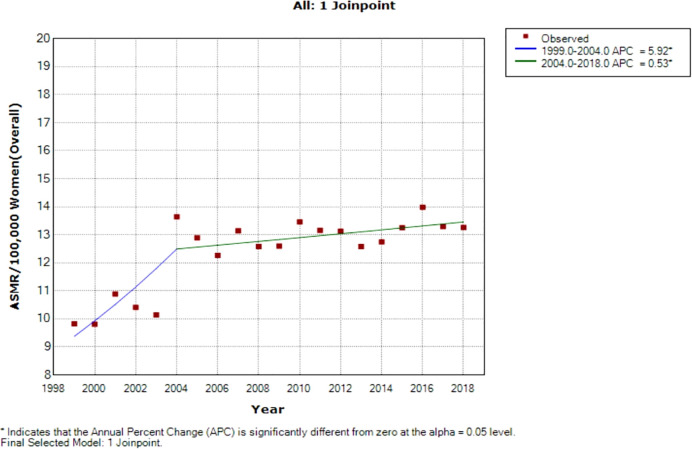
Joinpoint regression of national trends in age-standardized mortality rates of breast cancer in South Africa (1999–2018).

Joinpoint regression analysis of breast ASMR showed two trends: the first was a steep rise in ASMR at around 5.9% per annum from 1999 to 2004 (APC: 5.9%, P-value<0.001) and then a slow increase at 0.5% per annum from 2004 to 2018 (APC: 0.5%, P-value< 0.001) (**Figure 3**; **Table 2**).

**Table 2:** Joinpoint regression estimates of the trends in National and Ethnic age-standardized mortality rates of breast cancers in South Africa (1999–2018).

Cancer type	Trends	Year period		APC	95% CI		P-value	Comment
Breast								
Overall ASMR								
	1	1999–2004		5.9*	3.3	8.6	<0.001	Significant increase
	2	2004–2018		0.5*	0.1	1.0	<0.001	Significant increase
	Full range	1999–2018		1.4*	0.8	2.0	<0.001	Significant increase
Blacks								
	1	1999–2004		5.0*	0.9	9.3	<0.001	Significant increase
	2	2004–2018		3.0*	2.3	3.7	<0.001	Significant increase
	Full range	1999–2018		3.3*	2.7	4.0	<0.001	Significant increase
Indian/Asian								
	1	1999	2001	24.3	-20.0	92.9	0.3	Non-significant increase
	2	2001	2018	0.6	-0.6	1.9	0.3	Non-significant increase
	Full range	1999	2018	1.3*	0.2	2.5	< 0.001	Significant increase
Colored								
	1	1999	2002	-5.0	-18.1	10.0	0.4	Non-significant decrease
	2	2002	2005	12.9	-16.4	52.5	0.4	Non-significant increase
	3	2005	2008	-3.5	-27.1	27.7	0.8	Non-significant decrease
	4	2008	2018	3.4*	1.3	5.6	< 0.001	Significant increase
	Full range	1999	2018	2.7*	1.7	3.6	< 0.001	Significant increase
White								
	1	1999	2001	8.6	-9.3	30.1	0.3	Non-significant increase
	2	2001	2005	-1.9	-9.9	6.8	0.6	Non-significant decrease
	3	2005	2018	1.1*	0.2	2.0	< 0.001	significant increase
	Full range	1999	2018	0.7*	0.2	1.2	< 0.001	Significant increase

*Statistically significant p-value < 0.05.

### Ethnic trends of breast cancer mortality

**Figure 4:**
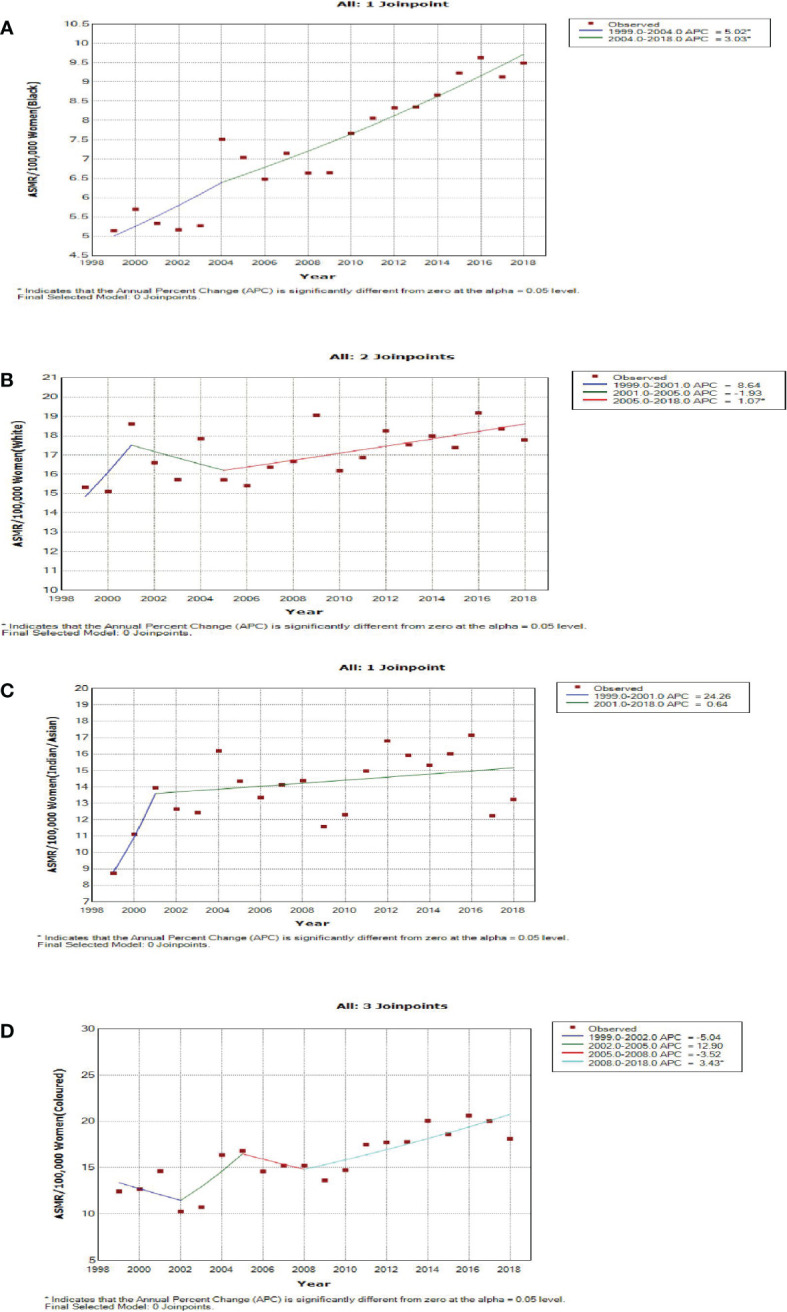
Joinpoint regression trends in age-standardized mortality rates of breast cancer in South Africa (1999–2018) for Black **(A)**, White **(B)**, Indian/Asian **(C)**, and Colored **(D)** ethnic groups.

Blacks followed by Whites had the highest annual number of breast cancer deaths throughout the study (**Figure 1A**; **Supplementary Table 1**). In 2018, the Colored ethnic group (18.11 per 100,000 women) had the highest breast cancer ASMR followed closely by the Whites (17.77/100,000 women) and Indian/Asians (13.24 per 100,000 women). The breast cancer ASMR among Blacks (9.49/100,000 women) was around half the rates among Coloreds or Whites in 2018 (**Figure 1B**; **Supplementary Table 1**). All the ethnic groups had varying increases in ASMR with Blacks (AAPC: 3.3%, P-value< 0.001) and Coloreds (AAPC: 2.7%, P-value< 0.001) having the highest increase, whereas Indian/Asians (AAPC: 1.3%, P-value< 0.001) and Whites (AAPC: 0.7%, P-value<0.001) had relatively lower increased rates. The joinpoint regression further showed that Blacks (APC: 5.0% vs. 3.0%) and Indian/Asians (APC: 24.3% vs. 0.6%) tended to have a reduction in the APC of breast ASMR between 2005 and 2018 as compared with the APC of the previous period (1999–2005), whereas Coloreds (APC: -3.5% vs. 3.4%) and Whites (APC: -1.9% vs. 1.1%) had higher annual rates of increase in the later periods of 2005–2018 (**Figures 1C**, **4A–D**; **Table 2**).

### Trends in mean age- and age-specific rates of breast cancer

In 2018, the mean age at death from breast cancer in South Africa was 59.98 ± 15.67 years and had been between 59 and 60 years during the study period (1999–2018) (**Table 2**). In 2018, the youngest mean age at death from breast cancer occurred among the Blacks (56.00 ± 15.36 years) whereas the average age at death among the Whites occurred around 11 years later (67.40 ± 15.28). Indian/Asians (63.94 ± 14.21 years) and Coloreds (60.63 ± 13.51 years) had a slightly lower mean age at death as compared with the Whites. The mean age at death from breast cancer slightly increased among all the ethnic groups (Whites: from 65 to 67 years; Indian/Asians: from 60 to 63 years; Coloreds: from 57 to 60 years; Blacks: from 55 to 56 years) over the study period (**Supplementary Table 1**).

### Age-specific death rates

**Figure 5:**
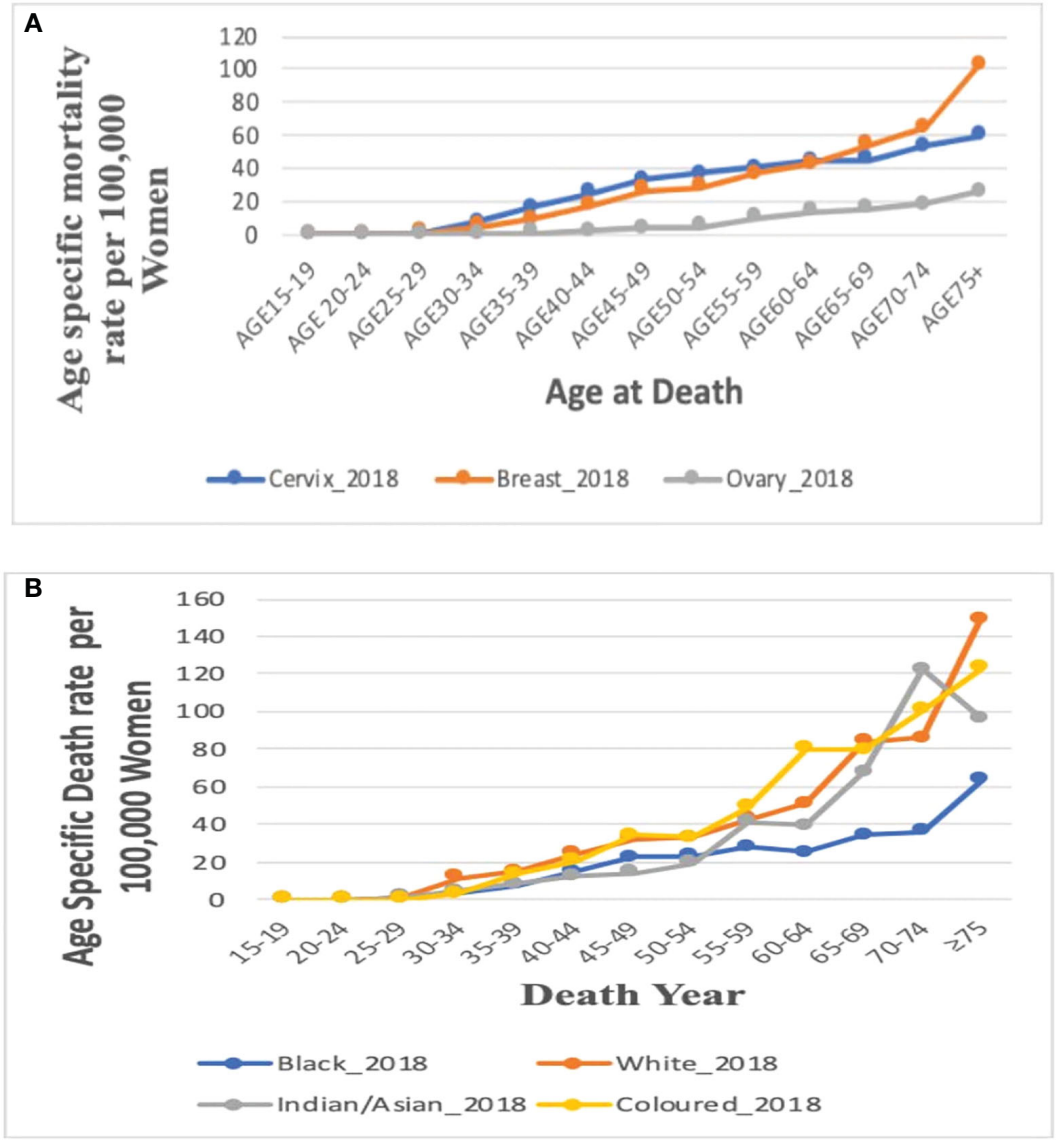
**(A)** Comparison of the overall age-specific death rates in 2018 in South Africa from breast, cervix, and ovaries. Figure 5 **(B)**. Age-specific death rate by ethnicity for breast cancer in 2018.

In 2018, the age-specific death rates of breast cancer increased with increasing age. Breast cancer mortality rates were lower than cervical cancer rates from 15 years till 60–64 years and then became the highest afterward to reach a peak at 75 years and above (**Figure 5A**; **Supplementary Table 2**).

### Age-specific death rate of breast cancer by ethnicity, 2018

In 2018, mortality rates of breast cancer increased with increasing age among all the four ethnic groups. Of the four ethnic groups, mortality was reported only among young Black women aged 15–24 years but the Black women had the lowest mortality rate from age 50 to 54 years. The Whites, Coloreds, and Indian/Asians had breast cancer mortality from 25 to 29 years, whereas Whites and Coloreds had the highest age-specific rates throughout all the age groups. The Indian/Asians had the lowest mortality rates till age 50–54 years, after which the rates increased and was close to the rates among Whites and Coloreds (**Figure 5B**; **Supplementary Table 2**).

### Joinpoint trends in the overall age-specific mortality rates of breast cancer, 1999–2018

**Figure 6:**
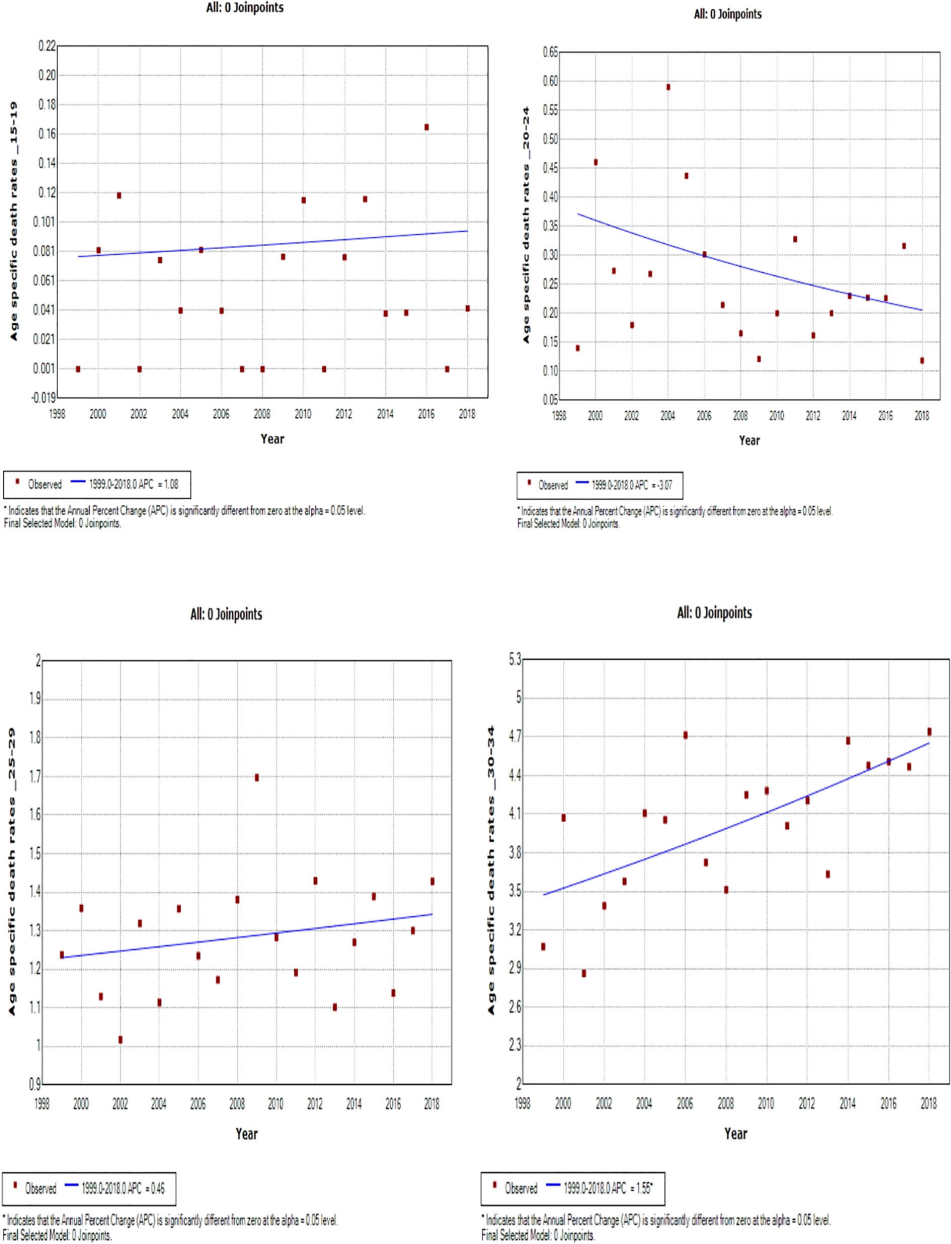
Joinpoint trends of age-specific death rates of breast cancer in South Africa. 1999–2018, among 19–34-year-old (5-year group).

**Figure 7:**
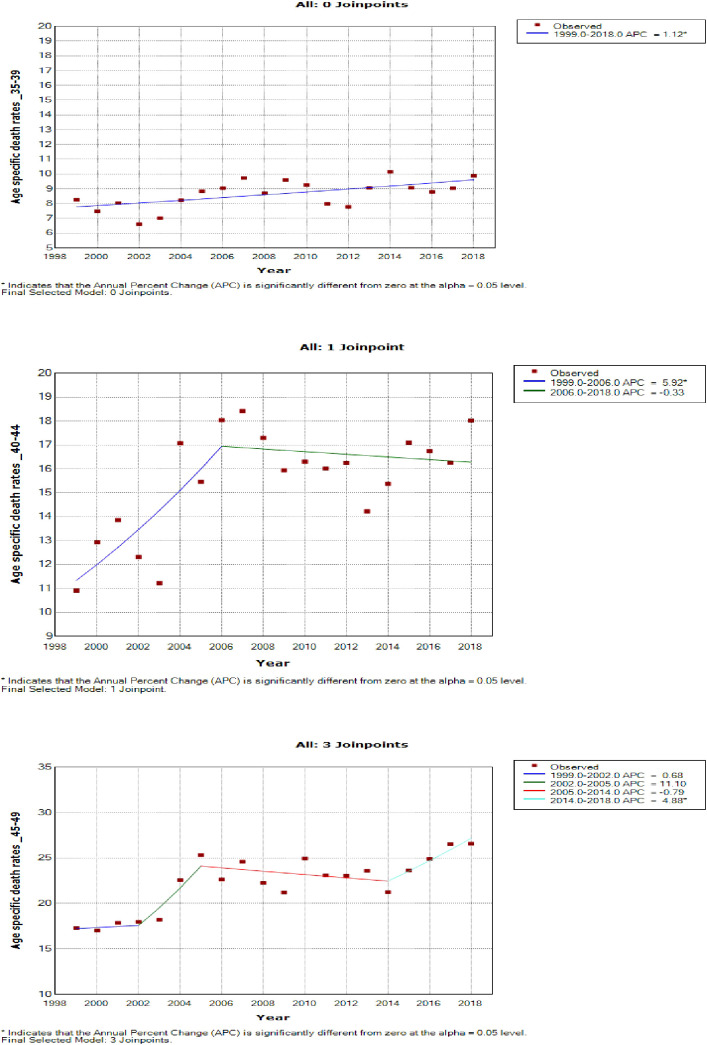
Joinpoint trends of age-specific death rates of breast cancer in South Africa, 1999–2018, among 35–49-year-olds (5-year group).

Young women aged 15–24 years had non-statistically significant breast cancer trends. Except for women aged 25–29 and 50–54 years who had stable trends (APC: 0.5%, P-value > 0.05), other women aged 30 years and older generally had an increased annual mortality rate of breast cancer (AAPC range: 0.5% to 2.0%, P-value< 0.05) from 1999 to 2018 (**Figures 6**, **7**; **Supplementary Table 3**).

**Figure 8:**
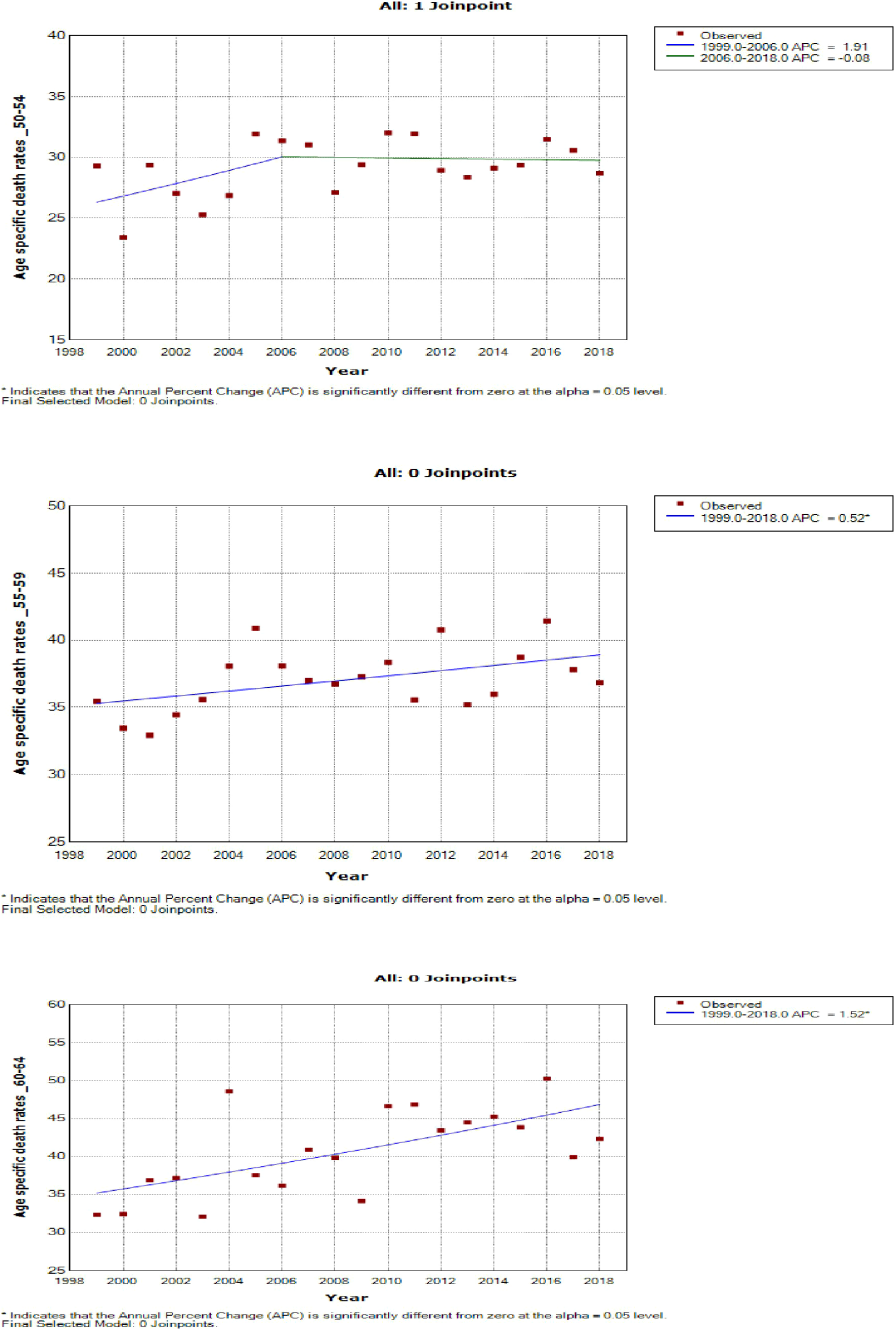
Joinpoint trends of age-specific death rates of breast cancer in South Africa, 1999–2018, among 50–64-year-olds (5-year group).

**Figure 9:**
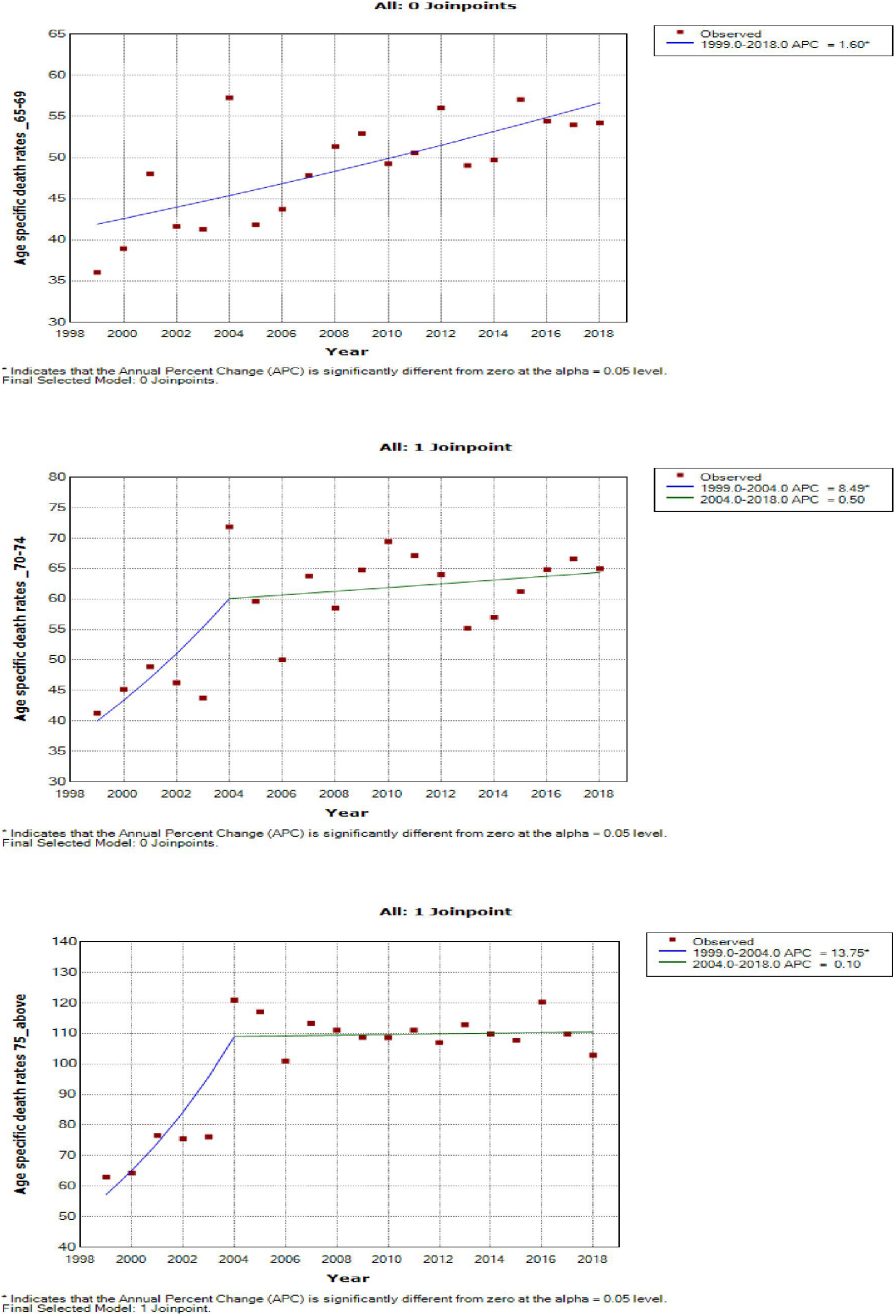
Joinpoint trends of age-specific death rates of breast cancer in South Africa, 1999–2018, among 65 years and above (5-year group).

Women aged 45–49 years had a rapid increase in mortality rates (APC: 4.9%, P-value< 0.001) between 2004 and 2018, whereas women aged 70 years and older had stable trends during a similar period (**Supplementary Table 3**; **Figures 7**–**9**).

### Joinpoint trends in the ethnic age-specific mortality rates of breast cancer, 1999–2018

Joinpoint regression modeling of the age-specific death rates of breast cancer revealed that Black teenagers (aged 15–19 yeas; AAPC: 0.6, P-value = 0.8) and young women aged 20–24 years (AAPC: -2.1, P-value = 0.3) respectively had nearly stable trends and a non-significant decline in breast cancer mortality rates from 1999 to 2018. All Black women aged 25 years and older had increased breast cancer death rates (AAPC range: 1.6% to 4.2%, P-value< 0.001) (**Supplementary Figure 1**; **Supplementary Table 3**).

There were few data points among young Whites, Indian/Asians, and Coloreds below 24 years. Thus, the conclusion from the joinpoint regression among these age groups was not reliable. From 1999 to 2018, there was a non-significant decline in breast cancer mortality rates among young Indian/Asians aged 25–34 years (AAPC -1.9 to -1.1, P-value >0.05) whereas there was a non-statistically significant rise in mortality rates among women aged 35–44 years (AAPC: 0.6 to 3.5, P-value > 0.05), 50–54 years (AAPC: 1.5, P-value = 0.3), 60 years and older (AAPC range: 1.0 to 2.0, P-value > 0.05), Indian/Asian aged 45–49 years (AAPC: -0.1, P-value = 1.0) (**Supplementary Figure 2**; **Supplementary Table 3**).

From 1999 to 2018, Colored women aged 25–44 years and 50–54 years (AAPC: 1.3 to 1.9, P-value >0.05) had non-statistically significant increased rates whereas women aged 45–49 and 55 years and older (APC range: 2.2 to 6.6, P-value< 0.001) had statistically significant increased mortality rates (**Supplementary Figure 3**; **Supplementary Table 3**). From 1999 to 2018, White women aged 30–44 years and those who were 75 years and older (APC range: 0.9%–5.6%, P-value< 0.001) had statistically significant increased mortality rates whereas Whites aged 50–69 years (AAPC: -0.3% to 0.6%, P-value >0.05) had approximately stable rates. However, Whites aged 45–49 (AAPC: 1.0%, P-value = 0.1) and 70–74 years (AAPC: 0.8%, P-value = 0.1) had non-significant increased rates. Young White women aged 25–29 years (AAPC: -2.1%, P-value = 0.3) and older women aged 50–54 (AAPC: -1.7%, P-value<0.001) respectively had a non-statistically significant decline (**Supplementary Figure 4**; **Supplementary Table 3**).

### Age period cohort analysis of overall and ethnic trends in breast cancer mortality

#### Local and net drift

**Figure 10:**
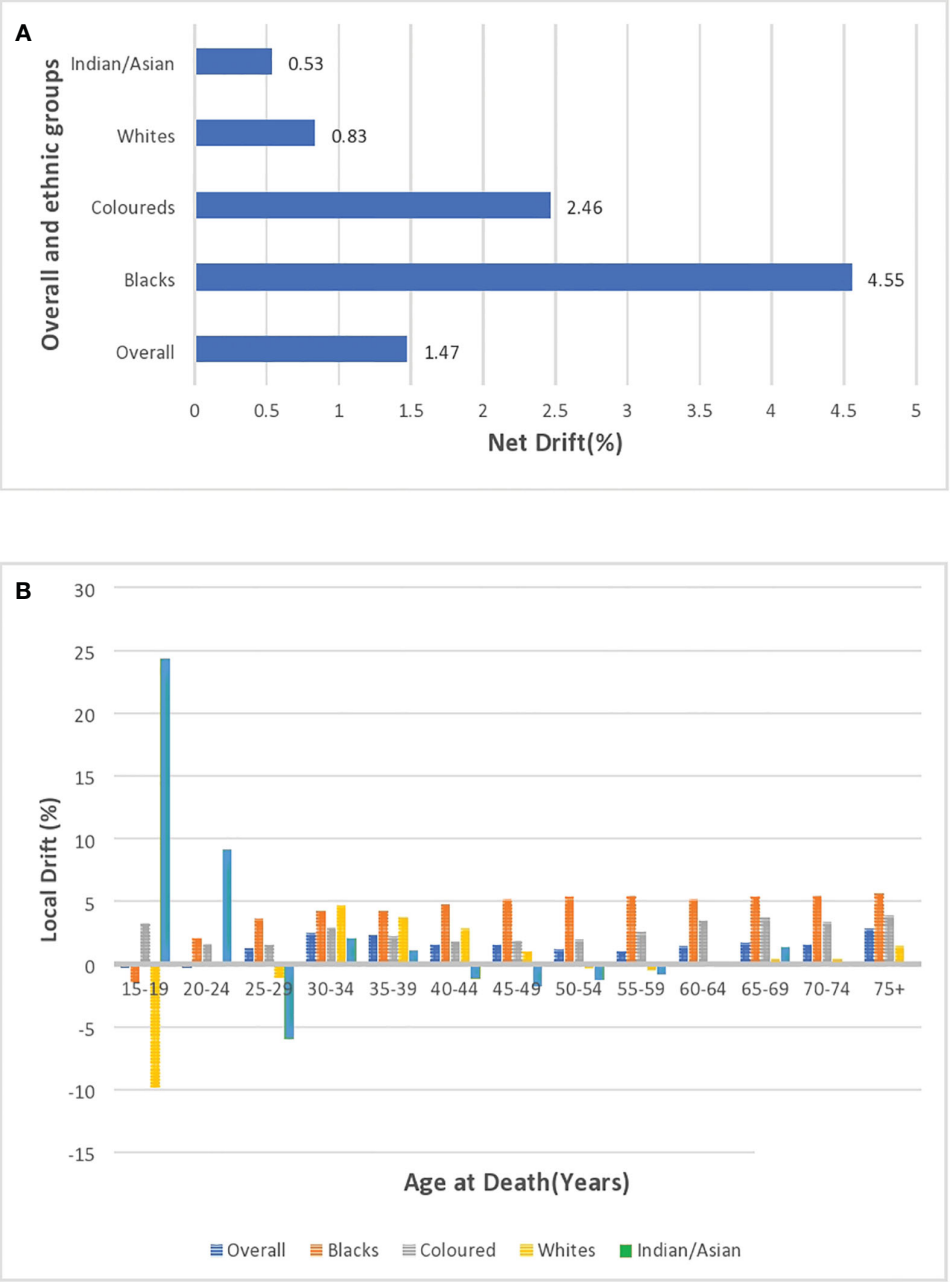
**(A)** Overall and ethnic net drifts of breast cancer mortality in South Africa (1999–2018). **(B)** Overall and ethnic local drifts of breast cancer mortality in South Africa (1999–2018).

After correcting for cohort and period effects, the overall net drift (similar to AAPC) of breast cancer mortality trends over the study period (1999–2018) was around 1.47% per annum (95% CI: 0.91%–2.04%). (**Figure 10A**; **Supplementary Table 4**). There was a positive net drift among all the ethnic groups with Blacks (4.55%, 95% CI: 3.94% to 5.16%) having the highest drift followed by the Coloreds (2.46%, 95% CI: 1.30% to 3.62%), Whites (0.83%, 95% CI: -1.13 to 2.82), and Indian/Asian (0.53%, 95% CI: -1.79 to 2.91) (**Supplementary Table 5**; **Figure 10A**). The net drifts of the trends in breast cancer mortality for the overall (P-value< 0.001), Blacks (P-value< 0.001), and Coloreds (P-value< 0.001) were statistically significant, whereas the net drifts for Whites (P-value = 0.41) and Indian/Asians (P-value = 0.66) were not statistically significant (**Table 3**).

**Table 3:** Wald Chi-square test for estimable functions of the age period cohort model in the overall and ethnic trends of breast cancer mortality in South Africa (1999–2018).

Cancer type	NetDrift = 0		All period RR = 1		All cohort RR = 1		All local drifts = net drift	
	Chi-square	P-value	Chi-square	P-value	Chi-square	P-value	Chi-square	P-value
Breast								
Overall	26.59	2.52E-07^*^	49.45	1.05E-10^*^	167.26	8.66E-28^*^	29.53	0.0055^*^
Black	222.87	2.14E^-50^*^^	298.27	2.36E^-64^*^^	658.99	1.01E^-130^*^^	9.55	0.73
White	0.68	0.41	2.34	0.50	69.68	5.09E^-09^*^^	45.54	1.70E^-05^*^^
Indian/Asian	0.20	0.66	0.83	0.84	12.93	0.61	12.62	0.48
Colored	17.70	2.58E^-05^*^^	25.80	1.05 E^-05^*^^	148.38	5.06E^-24^*^^	14.05	0.37

*Statistically significant at P-value< 0.05.

The overall local drift of breast cancer is<0 (although insignificant) for women younger than 25 years, and women older than 24 years had positive local drifts, with women aged 30–39 years and 75 above having drifts >2% (**Supplementary Table 4**; **Figure 10B**; **Supplementary Figure 5**). Young Blacks (<20 years) and Whites (<30 years) had insignificant local drifts below 0. However, Blacks (from 2.01% to 5.6%) and Coloreds (1.59% to 3.86%) generally had the highest positive local drifts that slightly increased with age from 20 years. Whites and Indian/Asians generally had low positive local drifts, but Whites aged 50–59 years and Indians/Asians aged 40–59 years had negative drifts (**Figure 10B**; **Supplementary Figures 6–9**; **Supplementary Table 5**).

#### Age effect

**Figure 11:**
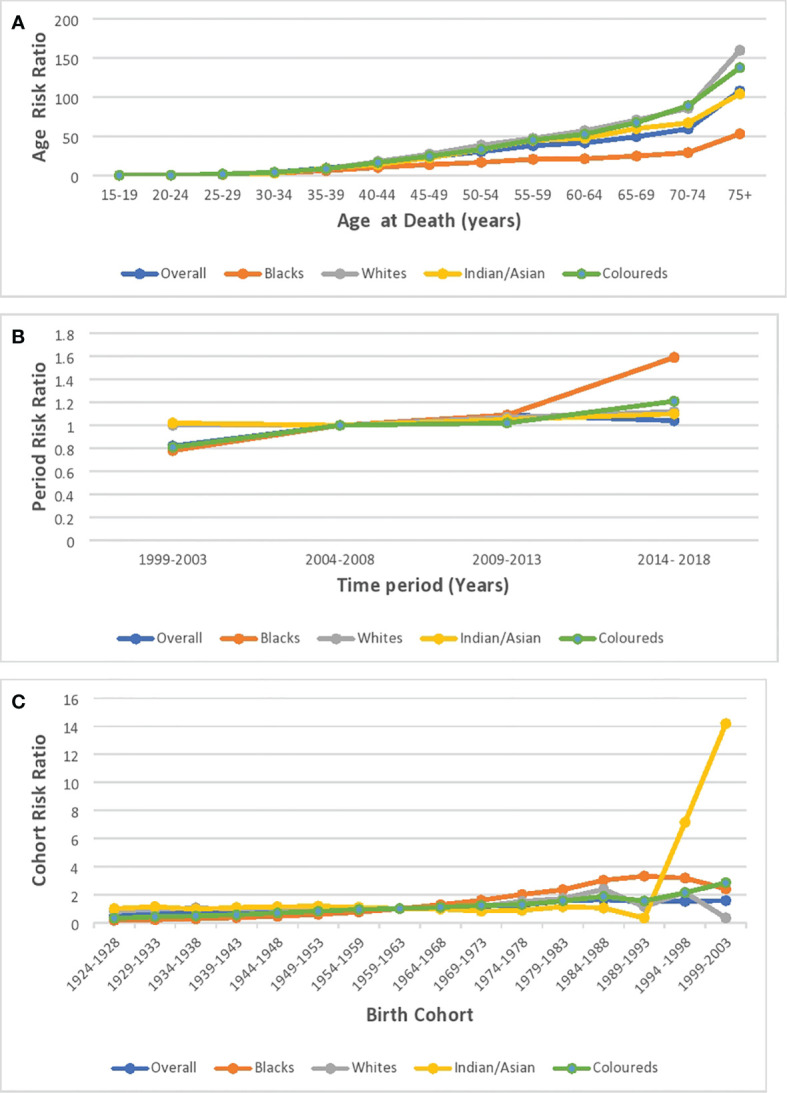
Ethnic and national breast cancer mortality risk ratio in South Africa due to **(A)** age, **(B)** period, and **(C)** cohort after the age–period–cohort analysis.

Based on the longitudinal/cross-sectional age curve, the relative risk (RR) of overall and ethnic breast cancer mortality increased with age, portraying a J-curve with a steep increase in risk from 70 years (**Figure 11A**; **Supplementary Figure 5**; **Supplementary Table 4**). Blacks (0.018, 95% CI: 0.008–0.042) and Indian/Asians (0.030, 95% CI: 0.001–1.664) had the least and second lowest RR at 15–19 years, but the Blacks’ RR (214.30 95% CI: 184.84–248.46 at >74 years) and Indian/Asians’ RR (116.83, 95% CI: 86.81–157.25 at >74 years), respectively, became the second highest and the least from 65 years. Coloreds (0.033, 95% CI: 0.003–0.315) and Whites (0.042, 95% CI: 0.002–0.730), respectively, had the third highest and highest RR at 15–19 years, but the Coloreds’ RR (283.04 95% CI: 239.51–334.49 at >74 years) and Whites’ RR (179.03, 95% CI: 157.22–203.87 at >74 years) became the highest and third highest from 45 years (**Supplementary Table 5**; **Figure 11A**; **Supplementary Figures 6–9**).

#### Period effect

The period RR for breast cancer mortality increased by around 18% from 1999–2003 to 2004–2008 (RR: 0.82, 95% CI:0.77–0.87) and then increased (although not significant) by 9% from 2004–2008 to 2009–2013 (RR: 1.09, 95% CI: 0.95–1.06). Subsequently, there was a 5% reduction in risk from 2009–2013 to 2014–2018 (RR: 1.04, 95% CI: 0.98–1.11) (**Figure 11B**; **Supplementary Figure 6**; **Supplementary Table 4**). All the ethnic groups had increased period RR from 1999 to 2018 with Blacks (RR: 1.52) having the highest increase between 2014 and 2018, followed by Coloreds (RR:1.21), Whites (RR:1.12), and Indians/Asians (RR:1.10) (**Figure 11B**; **Supplementary Table 5**; **Supplementary Figures 6–9**). The Wald’s test of the period effect was statistically significant for overall, Blacks, and Coloreds, but not significant among Whites and Indian/Asians (**Table 3**).

#### Cohort effect

The cohort RR of breast cancer mortality among those born during 1924–1928 (RR: 0.5, 95% CI: 0.43–0.57) was the least, and the risk increased among successive cohorts to a peak RR among those born between 1984 and 1988 (RR: 1.64, 95% CI: 1.35–1.99). Afterward, there was a decline in risk among successive birth cohorts to 1.57 among birth cohorts from 1999 to 2003 (**Figure 11C**; **Supplementary Figure 5**; **Supplementary Table 4**). With respect to ethnic cohort variations, the Blacks (RR: 0.16, 95% CI: 0.13–0.19) and the Coloreds (0.31, 95% CI: 0.25–0.40) had a relatively low RR among the cohort that were born between 1924 and 1928 and the risk increased among successive birth cohorts, with the cohort RR of Blacks becoming the highest among the 1964–1993 birth cohorts. The Whites (0.80, 95% CI: 0.69–0.93)) and Indian/Asians (1.00, 95% CI: 0.65–1.54) had a relatively higher cohort RR among the 1924–1928 birth cohorts, and there was minimal change in the mortality risk among their successive cohorts till the 1959–1963 cohort for Whites and 1984–1988 for Indian/Asians. Subsequently, the RR increased among Whites till those born in 1984–1988. While the mortality RR generally increased among successive Indian/Asian cohorts born between 1994 and 2003, the RR of similar birth cohorts of other ethnic groups reduced (**Figure 11C**; **Supplementary Figures 6–9**; **Supplementary Table 5**). The Wald’s test showed that the cohort effect was statistically significant for the overall trends and among all the ethnic groups (P-value< 0.001) except for the Indian/Asians (P-value = 0.61) (**Table 3**).

## Discussion

To our knowledge, this is the first study in SSA to utilize both joinpoint and A–P–C regression modeling techniques to evaluate the national trends in breast cancer mortality, stratified by ethnicity. This temporal trend analysis over 20 years (1999–2018) in South Africa is necessary to evaluate the impact of previous interventions and guide prioritization of health resources.

### Breast cancer mortality trends

The mortality rate of breast cancer in South Africa (13.27 per 100,000 women) was slightly lower than the average rates in Southern Africa (1), but higher than North American rates (1). We found that the mortality to incidence ratio (MIR) of breast cancer in South Africa (13.27 vs. 32.87 per 100,000 women, MIR:0.4) was slightly higher than the average of other Southern African Countries (15.6 vs. 46.2 per 100,000 women, MIR:0.34) and the North American region (12.6 vs. 84.8 per 100,000 women, MIR:0.15) but lower than the average MIR among Western African countries (17.8 vs. 37.3 per 100,000 women, MIR: 0.48) (30, 31).

As reported globally, we observed a significant period effect on the trends of breast cancer mortality in South Africa (27, 28). The net drift and joinpoint regression model indicated a rise in the breast cancer mortality rate by around 1.47% and 1.40% per annum from 1999 to 2018, respectively. Similarly, most countries in SSA and Asia had increasing mortality trends (29, 32, 33). In contrast, there was a decline in breast cancer mortality in most HICs and middle-income countries because of mass screening with mammography and clinical breast examination, hereditary screening of high-risk individuals, molecular and histopathological classification, early patient presentation, and prompt treatment with surgery (mastectomy), adjuvant hormone therapy, chemotherapy, and radiotherapy (32–37). The period RR and segmental APC suggest a reduction in the increasing rate of breast cancer mortality during the later period of 2004 to 2018 (5.9% per annum vs. 0.5% per annum). The apparent reduction in APC from 2004 may be partly explained by the reported decline in incidence from 1999 to 2010 (11). After the commencement of the multiracial democratic government in 1994 to replace the apartheid regime, policies were directed at expanding access to free reproductive public health services for majority of the previously marginalized population (especially the Black and Colored population) (9, 11, 38, 39). Furthermore, public enlightenment campaigns, ongoing multiple international and national breast cancer research, some screening programs by non-governmental organizations, and reduction of barriers to definitive care are also contributory to the reduced period RR in the later years (11, 40, 41). Since South Africa has one of the highest global HIV prevalences, HIV was hitherto a major competing risk of death in the country (20, 42, 43). Thus, the nationwide rollout of free ART in 2004 can partly explain the reduction in breast cancer mortality rate from 2005 (7, 38, 44).

### Age effect of breast cancer mortality trends

In line with previous studies, we found that there was a strong age effect on the breast cancer mortality trends as there was increased risks and rates with increasing age (27, 28, 42). Thus, it can be deduced that biological effect played a role in breast cancer mortality (27, 28, 42). The age effect was also apparent as the annual crude mortality rate of breast cancer was higher than the age-standardized rate among Whites over the study period from 1999 to 2018. South African women aged 30 years and older generally had an increased mortality rate, with that of women aged 30–39 and 75 years and above having a rapid rise (local drift >2%). Furthermore, we found that in the later years (2014–2018), women aged 45–49 years had a rapid rise in breast cancer mortality rate of around 5% per annum. Thus, death from premenopausal breast cancer is fast becoming a major cause of cancer deaths among young South Africans and globally (4, 34, 43, 45, 46). Nonetheless, a decline in mortality rate occurred despite increased incidence among young women in some countries in LMICs and HICs and this was attributed to routine and opportunistic screening during antenatal and contraceptive consultations and a strong cohort effect from the 1950s (45–47).

In comparison with postmenopausal breast cancer, the premenopausal breast cancer type is usually more aggressive, advanced-stage at diagnosis, triple-negative, and with worse prognosis (36, 48). Remarkably, the screening, diagnosis, and treatment of premenopausal breast cancer are a challenge because dense breast tissue of young women may obfuscate pathological tissues (4, 43). Obesity appears to be the major driver of sporadic postmenopausal breast cancer (4, 49).

Notably, South African women aged 50–59 years had nearly stable mortality trends (AAPC = 0.5%). Similarly, women aged 50–69 years had the lowest AAPC in Sub-Saharan Africa (AAPC:<50 years: 0.53%, 50–69 years: 0.34%, ≥70 years: 0.82%) and in all other global regions from 1990 to 2017 (48). This pattern suggests that women aged 50–59 years generally had routine or opportunistic screening, or there was reduction in perimenopausal HRT use from 2000 (4, 34, 45).

The increased mortality rate of breast cancer among South African women older than 70 years may be associated with increasing life expectancy and comorbid factors (33, 34, 36, 48, 50). Thus, research and public health interventions aimed at reducing the burden of breast cancer should target all age groups from 25 years.

### Cohort effect of breast cancer mortality

We reported a rise in RR of breast cancer mortality among South African birth cohorts from 1924 to 1988 (0.5 to 1.64) and a subsequent decline among recent cohorts from 1988 to 2003. A decline in cohort mortality risks around 1920 and 1960 in USA and East Asia, respectively, was previously reported (27, 28, 51, 52). Each successive South African birth cohort experienced increased risk factors (leading to mortality) of breast cancer such as increased prevalence of obesity, smoking, alcohol consumption, low fertility rate, prolonged use of hormonal contraceptive, late age at first pregnancy (usually on account of prolonged years of education), reduced period of breastfeeding (mainly because of pressures of work), and westernization of diet (increased fatty and high-protein diet) (5, 6, 11, 36, 53–56). However, the improved socioeconomic status, educational attainment, health seeking behavior, reduction in smoking prevalence, and increased awareness and access to healthcare among recent South African cohorts especially after the commencement of the multiracial democracy in 1994 might partly explain the reduction in breast cancer cohort mortality risk from 1988 (10, 45, 57, 58).

### Ethnic disparity of breast cancer trends

We observed that ethnic disparity in breast cancer mortality in South as Blacks had around half the breast cancer mortality rates of Colored, Whites, and Indian/Asians. In contrast, Blacks had higher breast cancer mortality rates as compared with Whites in USA (35, 59, 60).

Our analysis showed that breast cancer mortality RR increased among successive Black and Colored cohorts from 1924 to 2003 and Blacks had the highest mortality risk among recent cohorts (1974–2003). Historically, Blacks had protective breast cancer risk factors such as increased prevalence of late menarche, early age at first birth (Black South African culture supports early/teenage pregnancy), high parity, prolonged breastfeeding practices, and high-fiber diet (6, 27, 60). However, successive cohorts of Blacks and Coloreds had experienced increased westernized diet, increased use of hormonal contraceptives, decreased fertility rate, and increased prevalence of obesity and sedentary jobs (5, 55, 56, 61). Furthermore, successive cohorts of Coloreds also had increased prevalence of smoking and alcohol rates (62–64). Despite having the lowest breast cancer incidence, Blacks (9.49 vs. 19.32 per 100,000 MIR: 0.49) and Coloreds (18.11 vs. 47.9 per 100,000 women, MIR: 0.38) had the highest MIR as compared with Indian/Asians (13.24 vs. 15.24 per 100,000 women, MIR: 0.26) and Whites (17.77 vs. 84.49 per 100,000 women, MIR:0.21), which suggests a worst survival rate (30, 64, 65). During the apartheid era, successive cohorts of Blacks and Coloreds had worse breast cancer survival because they usually have poor awareness, advanced staged cancer, and poor access to healthcare especially among rural dwellers (6, 38, 66). Furthermore, Blacks usually have an aggressive, premenopausal, and triple-negative form of breast cancer (6). Our study showed that the expected cohort RR decline of breast cancer after the expansion of access to healthcare since the commencement of the multiracial democracy in 1994 has not occurred (9, 38, 57).

The earliest White and Indian/Asian birth cohorts had relatively high mortality RR, which was nearly stable until 1969–1973 when the RR among Whites slightly increased whereas that of Indian/Asian cohorts declined. The increased mortality risk among the recent White cohorts may be driven by modifiable factors such as obesity, smoking, alcohol consumption, and use of hormonal contraceptives, whereas the decline among Indian/Asians is similar to the cohort trends in East Asia and America that was attributable to improved awareness, screening, and improved healthcare (27, 52, 62).

The period effect of breast cancer mortality from 1999 to 2018 was noted among Blacks and Coloreds, with rapid drifts of 4.6% and 2.5% per annum, respectively. The improved socioeconomic status and shift in reproductive behaviors without commensurate access to screening and oncological care among Blacks and Coloreds led to increased breast cancer mortality from 1999 to 2018. Nonetheless, a reduced acceleration among Blacks (APC: 5.0% vs. 3.0%), in the later period (2004–2018), may be attributed to some improvement in public reproductive health and oncological services (9–11, 38, 52, 57). Furthermore, the national rollout of free ART in 2004 partly contributed to death reduction among Black HIV-positive breast cancer patients (38, 44, 67, 68). In contrast, Coloreds (APC: -3.5% vs. 3.4%) had increased breast cancer mortality rates in the later years (2008–2018), suggesting that the public health interventions are yet to impact breast cancer outcome among them. This may also suggest that the cohort and age effects are stronger than the period effect among them.

The period effect of breast cancer mortality was not statistically significant among Whites and Indian/Asians, and they had low net drifts (Whites, 0.83%; Indian/Asian, 0.53%). Indeed, Whites and Indian/Asians had access to private health facility with optimum oncological facilities that are comparable with healthcare services in HICs (11, 57). Thus, sociopolitical and public health interventions by the South African government may not impact on the outcome of breast cancer care among them. However, this study highlighted an increased breast cancer mortality among Whites (APC: -1.9% vs. 1.1%) in the later years (2008–2018), which calls for further research. The apparent decline in mortality rate among Indian/Asians can be attributed to early diagnosis and treatment (27, 52).

Blacks had the youngest average age at death (56 years) followed by Coloreds (60.6 years), Indian/Asian (63.9 years), and White (67.4 years), suggesting that worst survival or more premenopausal deaths occurred among them. The observed rise in breast cancer mortality among the Whites in the later years may be occurring at old age. Indeed, the CMR of breast cancer was around thrice the ASMR among Whites, suggesting that majority of the deaths occurred in the elderly. Strikingly, women aged 25–39 years generally had the highest rise in breast cancer mortality. Remarkably, deaths from premenopausal breast cancer (<45 years) increased whereas postmenopausal breast cancer deaths declined among Coloreds, possibly suggesting increased modifiable risks and poor access to screening and early care among young Coloreds. The negative drifts among Whites aged 50–69 years and Indian/Asians aged 40–59 years may suggest that women of the two ethnic groups commenced screening at 40–50 years according to international guidelines (27, 33, 47, 69). There is a need for public enlightenment campaigns, modifiable risk reduction, and provision of optimum screening and treatment modalities among women of all ethnic groups over 20 years.

## Strength and limitation

We utilized national mortality data that have been adjudged to be of high quality to comprehensively evaluate the trends in breast cancer mortality, based on the A–P–C and joinpoint regression models to unmask cues and information toward control of breast cancer in South Africa (17, 20).

One limitation of this study was the missing information on the stage and histological types of the cancers that can further improve the interpretation of our results (7, 70). Furthermore, there may be some underreporting of deaths that occurred outside health institutions. However, it is mandatory to report all deaths to the Department of Home Affairs before burial. Since our study was population based, we exercised caution while interpreting at the individual level to avoid the risk of ecological fallacy (71).

## Conclusion

In conclusion, we found that a significant age period cohort effect was observed for breast cancer mortality trends. There was a breast cancer rise of around 1.5% per annum from 1999 to 2018, largely driven by a rapid rise in deaths among young women (in the last 10 years of the study). The breast cancer mortality risk increased from the early cohorts but started decreasing among the recent cohorts born from 1989 to 2003. The period effect from screening and expansion of healthcare services after multiracial democracy in 1994 only led to minimal reduction in deaths of breast cancer, especially among women aged 50–59 years. The breast cancer mortality rate among Blacks was around half of the rates among the non-Blacks. Each of the four ethnic groups had differential trends and burden on account of peculiar socioeconomic, cultural, screening behavior, access to optimum care, awareness, and sexual and reproductive behavior. The identified disparities and trends are very useful for designing targeted intervention.

## Brief policy implications

The South African government launched the national breast program in 2017 to promote prevention and early detection and prompt optimum treatment of breast (72). Such initiative is expected to reverse the current increasing burden of the breast cancer deaths. However, based on the results of our study, we recommend that screening for breast cancer should be intensified in all age groups, as our results suggest strong/highest risks among young cohorts. In contrast to screening policies of HICs, population-based routine screening of breast cancer with mammography was not recommended in the South African guideline, largely on account of cost to the health system (72). However, we recommend that in addition to the current recommendation of promoting awareness, regular clinical breast examination, prompt treatment, and mammography should be considered especially commencing at a young age, possibly 35–40 years, as majority of breast cancer cases are sporadic (4). Opportunistic mammography can also be encouraged as part of routine occupational medical examination (28). Women that can afford mammography should also be offered pending when the health system can provide it for all women.

Since we found that cohort effect is a major driver of breast cancer mortality trends in the country, primary prevention should target all the known risk factors of breast cancer such as reduction of obesity, promoting breastfeeding, and reproductive behaviors. Breast cancer prevention should be part of the social marketing for promoting the cessation of tobacco smoking and alcohol consumption (4, 28). Interventions that target ethnic burden of breast cancer mortality can be considered. Indeed, older White women had higher burden of breast cancer mortality and targeted intervention will be necessary.

## Data availability statement

Publicly available datasets were analyzed in this study. This data can be found here: The Statistics South Africa website.

## Ethics statement

The studies involving human participants were reviewed and approved by Human Research and Ethics Committee (Medical) of the University of the Witwatersrand (Clearance certificate number: M190544). Written informed consent for participation was not required for this study in accordance with the national legislation and the institutional requirements.

## Author contributions

Conceptualization and study design: GO, EM, EL, OCE; data acquisition: GO, EM, EL; data management and data analysis: GO; data interpretation: GO, EM, EL, OCE; writing original draft: GO; critical review of manuscript and acceptance on the manuscript submission: GO, EM, EL, OCE; supervision of the project: EM, EL, OCE. All authors contributed to the article and approved the submitted version
