# Supplementary material for: Population-based temporal trends and ethnic disparity in breast cancer mortality in South Africa (1999-2018): Joinpoint and age–period–cohort regression analyses
Source: Front Oncol. 2023 Feb 3;13:1056609. doi: 10.3389/fonc.2023.1056609 (PMC9935608; doi:10.3389/fonc.2023.1056609)
Supplement: Supplementary file 1 [file Table_1.doc]

**Supplementary Table 1. Trends in the mortality rate and mean age at death of breast cancer among the ethnic groups of South Africa ,1999 – 2018**

| **Year** | **Black n= 23,431)** | | | | **White (n= 14,224)** | | | |  | **Coloured (n= 6,677)** | | | | **Indian/Asian (n= 1,983)** | | | |
| --- | --- | --- | --- | --- | --- | --- | --- | --- | --- | --- | --- | --- | --- | --- | --- | --- | --- |
|  | **Mortality (% of gynae & breast)**  **≥ 15 year** | **Age**  **(mean ± SD)** | **CMR** | **ASMR** | **Mortality (% of gynae & breast)**  **≥ 15 years** | **Age**  **mean ± SD** | **CMR** | **ASMR** |  | **Mortality (% of gynae & breast)**  **≥ 15 years** | **Age**  **mean ± SD** | **CMR** | **ASMR** | **Mortality (% of gynae & breast)**  **≥ 15 years** | **Age**  **mean ± SD** | **CMR** | **ASMR** |
| 1999 | 602 | 55.41±15.48 | 5.25 | 5.14 | 527 | 65.04±14.74 | 29.06 | 15.32 |  | 199 | 57.33±16.12 | 14.40 | 12.44 | 44 | 60.14±11.91 | 10.45 | 8.72 |
| 2000 | 708 | 54.70±15.88 | 5.97 | 5.70 | 525 | 65.91±15.44 | 28.95 | 15.10 |  | 205 | 58.40±15.00 | 14.42 | 12.70 | 58 | 60.24±13.28 | 13.46 | 11.12 |
| 2001 | 751 | 55.94±15.47 | 6.02 | 5.33 | 642 | 65.39±14.90 | 35.41 | 18.60 |  | 241 | 57.67±14.16 | 16.50 | 14.62 | 75 | 59.44±12.09 | 17.02 | 13.95 |
| 2002 | 741 | 55.95±15.26 | 5.86 | 5.17 | 593 | 66.13±13.81 | 32.26 | 16.59 |  | 170 | 58.87±15.14 | 11.48 | 10.27 | 68 | 59.56±14.05 | 15.22 | 12.64 |
| 2003 | 805 | 55.53±15.75 | 6.10 | 5.27 | 574 | 67.24±14.66 | 31.66 | 15.71 |  | 189 | 58.33±14.04 | 12.32 | 10.73 | 74 | 58.73±11.85 | 16.14 | 12.42 |
| 2004 | 951 | 55.28±15.12 | 7.74 | 7.51 | 655 | 65.66±14.31 | 35.86 | 17.85 |  | 251 | 58.50±14.73 | 17.21 | 16.38 | 88 | 62.32±12.43 | 20.34 | 16.20 |
| 2005 | 970 | 55.69±15.29 | 7.78 | 7.04 | 636 | 66.95±14.11 | 34.77 | 15.70 |  | 299 | 57.96±14.11 | 19.79 | 16.81 | 91 | 59.90±13.11 | 20.41 | 14.35 |
| 2006 | 919 | 53.72±14.82 | 7.26 | 6.48 | 619 | 66.33±14.51 | 33.73 | 15.41 |  | 266 | 57.53±14.37 | 17.30 | 14.60 | 88 | 60.85±13.18 | 19.44 | 13.34 |
| 2007 | 1,029 | 56.44±15.94 | 8.01 | 7.15 | 675 | 66.91±13.77 | 36.67 | 16.37 |  | 285 | 58.46±14.22 | 18.77 | 15.22 | 95 | 59.15±13.50 | 20.69 | 14.13 |
| 2008 | 977 | 55.94±15.21 | 7.25 | 6.64 | 699 | 66.47±14.53 | 36.85 | 16.68 |  | 305 | 57.33±14.55 | 18.52 | 15.23 | 99 | 62.01±13.74 | 20.38 | 14.38 |
| 2009 | 1,006 | 55.33±15.29 | 7.34 | 6.65 | 703 | 66.43±14.53 | 38.47 | 19.06 |  | 279 | 59.01±14.46 | 16.70 | 13.64 | 85 | 60.87±11.75 | 17.00 | 11.59 |
| 2010 | 1,144 | 55.34±14.81 | 8.27 | 7.67 | 710 | 66.75±14.29 | 36.67 | 16.18 |  | 310 | 58.87±13.25 | 18.36 | 14.73 | 94 | 60.44±13.07 | 18.45 | 12.31 |
| 2011 | 1,282 | 56.83±15.07 | 9.12 | 8.06 | 748 | 67.52±13.87 | 38.71 | 16.87 |  | 378 | 58.63±14.26 | 21.87 | 17.49 | 111 | 62.80±12.66 | 21.91 | 14.97 |
| 2012 | 1,349 | 56.42±15.33 | 9.48 | 8.32 | 795 | 66.23±14.02 | 40.64 | 18.25 |  | 382 | 59.68±13.93 | 21.83 | 17.74 | 127 | 62.36±12.85 | 24.76 | 16.80 |
| 2013 | 1,491 | 56.32±14.91 | 9.83 | 8.35 | 792 | 67.02±14.49 | 40.42 | 17.52 |  | 412 | 60.19±14.39 | 22.72 | 17.80 | 124 | 60.65±13.10 | 23.96 | 15.92 |
| 2014 | 1,593 | 56.35±15.25 | 10.51 | 8.66 | 826 | 67.67±14.53 | 42.55 | 17.97 |  | 459 | 59.68±14.26 | 25.23 | 20.07 | 120 | 61.15±13.64 | 23.02 | 15.33 |
| 2015 | 1,676 | 56.49±15.24 | 10.82 | 9.23 | 822 | 67.71±14.25 | 42.31 | 17.38 |  | 469 | 59.91±14.18 | 25.24 | 18.61 | 137 | 62.90±13.04 | 25.78 | 16.02 |
| 2016 | 1,791 | 56.25±15.06 | 11.27 | 9.62 | 909 | 67.74±14.05 | 46.89 | 19.17 |  | 508 | 60.31±14.28 | 27.49 | 20.63 | 151 | 65.07±13.32 | 27.91 | 17.16 |
| 2017 | 1,744 | 55.99±15.03 | 10.78 | 9.13 | 886 | 67.70±14.46 | 45.58 | 18.35 |  | 541 | 60.70±14.71 | 28.84 | 20.04 | 115 | 63.02±15.35 | 20.75 | 12.24 |
| 2018 | 1,902 | 56.00±15.36 | 11.48 | 9.49 | 888 | 67.40±15.28 | 45.13 | 17.77 |  | 529 | 60.63±13.51 | 27.37 | 18.11 | 139 | 63.94±14.21 | 23.89 | 13.24 |

Supplementary Table 2 The overall and ethnic age specific mortality rates of breast cancers and in South Africa, 2018

| **Age (Year)** | **15-19** | **20-24** | **25-29** | **30-34** | **35-39** | **40-44** | **45-49** | **50-54** | **55-59** | **60-64** | **65-69** | **70-74** | **≥75** |
| --- | --- | --- | --- | --- | --- | --- | --- | --- | --- | --- | --- | --- | --- |
| **Breast** | | | | | | | | | | | | | |
| Overall | 0.04 | 0.11 | 1.42 | 4.74 | 9.89 | 18.02 | 26.57 | 28.70 | 36.84 | 42.31 | 54.19 | 64.99 | 102.88 |
| Black | 0.05 | 0.14 | 1.42 | 4.01 | 8.30 | 14.99 | 22.78 | 23.66 | 27.76 | 25.64 | 34.43 | 36.43 | 63.57 |
| White | 0.00 | 0.00 | 0.75 | 12.07 | 14.84 | 24.96 | 32.30 | 33.20 | 43.05 | 51.11 | 84.09 | 86.42 | 149.26 |
| Indian/Asian | 0.00 | 0.00 | 1.74 | 4.84 | 8.55 | 12.87 | 14.49 | 19.87 | 41.74 | 39.84 | 67.75 | 122.14 | 96.44 |
| Coloured | 0.00 | 0.00 | 0.46 | 3.44 | 14.07 | 21.08 | 34.47 | 32.92 | 49.02 | 80.04 | 79.82 | 101.04 | 123.17 |

**Supplementary Table 3. Join point estimates of the national and ethnic trends in age specific mortality rates for breast cancers South Africa, 1999 – 2018**

| **Breast** | **Range** | **Lower Endpoint** | **Upper Endpoint** | **AAPC** | **Lower CI** | **Upper CI** | **Test Statistic** | **P-Value** | **Comment** |
| --- | --- | --- | --- | --- | --- | --- | --- | --- | --- |
| **Overall** |  |  |  |  |  |  |  |  |  |
| 15-19 | Full Range | 1999 | 2018 | 1.1 | -3.6 | 6.0 | 0.5 | 0.6 | Not statistically significant |
| 20-24 | Full Range | 1999 | 2018 | -3.1 | -6.4 | 0.4 | -1.8 | 0.1 | Not statistically significant |
| 25-29 | Full Range | 1999 | 2018 | 0.5 | -0.5 | 1.5 | 1.0 | 0.3 | Stable |
| 30-34 | Full Range | 1999 | 2018 | 1.6* | 0.7 | 2.4 | 3.9 | <0.001 | Statistically significant increased trend |
| 35-39 | Full Range | 1999 | 2018 | 1.1* | 0.4 | 1.9 | 3.2 | <0.001 | Statistically significant increased trend |
| 40-44 | 1 | 1999 | 2006 | 5.9* | 1.7 | 10.3 | 3.0 | 0.0 | Statistically significant increased trend |
|  | 2 | 2006 | 2018 | -0.3 | -1.9 | 1.3 | -0.4 | 0.7 | Stable |
|  | Full Range | 1999 | 2018 | 1.4* | 0.4 | 2.5 | 2.9 | <0.001 | Statistically significant increased trend |
| 45-49 | 1 | 1999 | 2002 | 0.7 | -7.8 | 9.9 | 0.2 | 0.9 | Not statistically significant |
|  | 2 | 2002 | 2005 | 11.1 | -5.7 | 30.9 | 1.4 | 0.2 | Not statistically significant |
|  | 3 | 2005 | 2014 | -0.8 | -2.4 | 0.9 | -1.1 | 0.3 | Not statistically significant |
|  | 4 | 2014 | 2018 | 4.9* | 0.1 | 9.9 | 2.3 | <0.001 | Statistically significant increased trend |
|  | Full Range | 1999 | 2018 | 1.8* | 1.1 | 2.6 | 5.1 | <0.001 | Statistically significant increased trend |
| 50-54 | 1 | 1999 | 2006 | 1.9 | -1.2 | 5.1 | 1.3 | 0.2 | Not statistically significant |
|  | 2 | 2006 | 2018 | -0.1 | -1.4 | 1.3 | -0.1 | 0.9 | Stable |
|  | Full Range | 1999 | 2018 | 0.5 | -0.1 | 1.2 | 1.8 | 0.1 | Stable |
| 55-59 | Full Range | 1999 | 2018 | 0.5* | 0.0 | 1.0 | 2.3 | <0.001 | Statistically significant increased trend |
| 60-64 | Full Range | 1999 | 2018 | 1.5* | 0.6 | 2.4 | 3.5 | <0.001 | Statistically significant increased trend |
| 65-69 | 1 | 1999 | 2001 | 12.7 | -16.5 | 52.2 | 0.9 | 0.4 | Not statistically significant |
|  | 2 | 2001 | 2018 | 1.2* | 0.3 | 2.2 | 2.8 | <0.001 | Statistically significant increased trend |
|  | Full Range | 1999 | 2018 | 1.6* | 0.8 | 2.4 | 4.4 | <0.001 | Statistically significant increased trend |
| 70-74 | 1 | 1999 | 2004 | 8.5* | 1.7 | 15.7 | 2.7 | 0.0 | Statistically significant increased trend |
|  | 2 | 2004 | 2018 | 0.5 | -0.6 | 1.6 | 0.9 | 0.4 | Stable |
|  | Full Range | 1999 | 2018 | 1.7* | 0.6 | 2.8 | 3.4 | <0.001 | Statistically significant increased trend |
| 75+ | 1 | 1999 | 2004 | 13.8* | 9.3 | 18.4 | 6.9 | <0.001 | Statistically significant increased trend |
|  | 2 | 2004 | 2018 | 0.1 | -0.6 | 0.8 | 0.3 | 0.8 | Stable |
|  | Full Range | 1999 | 2018 | 2.0* | 0.8 | 3.3 | 3.3 | <0.001 | Statistically significant increased trend |
| **Blacks** |  |  |  |  |  |  |  |  |  |
| 15-19 | Full Range | 1999 | 2018 | 0.6 | -3.8 | 5.2 | 0.3 | 0.8 | Not statistically significant |
| 20-24 | Full Range | 1999 | 2018 | -2.0 | -5.5 | 1.6 | -1.2 | 0.3 | Not statistically significant |
| 25-29 | 1 | 1999 | 2003 | 3.0 | -11.3 | 19.5 | 0.4 | 0.7 | Not statistically significant |
|  | 2 | 2003 | 2010 | -2.3 | -9.3 | 5.3 | -0.7 | 0.5 | Not statistically significant |
|  | 3 | 2010 | 2018 | 5.8* | 1.1 | 10.6 | 2.7 | <0.001 | Statistically significant increased trend |
|  | Full Range | 1999 | 2018 | 1.6* | 0.3 | 2.9 | 2.6 | <0.001 | Statistically significant increased trend |
| 30-34 | Full Range | 1999 | 2018 | 3.3* | 2.3 | 4.4 | 6.6 | <0.001 | Statistically significant increased trend |
| 35-39 | 1 | 1999 | 2007 | 4.1* | 1.1 | 7.2 | 3.0 | <0.001 | Statistically significant increased trend |
|  | 2 | 2007 | 2011 | -1.0 | -12.9 | 12.5 | -0.2 | 0.9 | Not statistically significant |
|  | 3 | 2011 | 2018 | 3.4* | 0.1 | 6.7 | 2.3 | <0.001 | Statistically significant increased trend |
|  | Full Range | 1999 | 2018 | 2.3* | 1.5 | 3.1 | 6.4 | <0.001 | Statistically significant increased trend |
| 40-44 | 1 | 1999 | 2006 | 8.9* | 3.1 | 14.9 | 3.4 | <0.001 | Statistically significant increased trend |
|  | 2 | 2006 | 2009 | -5.3 | -34.6 | 37.1 | -0.3 | 0.8 | Not statistically significant |
|  | 3 | 2009 | 2018 | 3.3* | 0.1 | 6.6 | 2.3 | <0.001 | Statistically significant increased trend |
|  | Full Range | 1999 | 2018 | 2.8* | 1.6 | 4.0 | 4.9 | <0.001 | Statistically significant increased trend |
| 45-49 | 1 | 1999 | 2005 | 10.1* | 4.5 | 16.0 | 3.9 | <0.001 | Statistically significant increased trend |
|  | 2 | 2005 | 2018 | 2.8* | 1.5 | 4.1 | 4.7 | <0.001 | Statistically significant increased trend |
|  | Full Range | 1999 | 2018 | 4.2* | 3.2 | 5.2 | 9.1 | <0.001 | Statistically significant increased trend |
| 50-54 | 1 | 1999 | 2002 | 0.1 | -16.7 | 20.2 | 0.0 | 1.0 | Stable |
|  | 2 | 2002 | 2005 | 10.3 | -22.4 | 56.9 | 0.6 | 0.6 | Not statistically significant |
|  | 3 | 2005 | 2018 | 2.7* | 1.1 | 4.3 | 3.6 | <0.001 | Statistically significant increased trend |
|  | Full Range | 1999 | 2018 | 3.6* | 2.7 | 4.5 | 8.2 | <0.001 | Statistically significant increased trend |
| 55-59 | Full Range | 1999 | 2018 | 3.1* | 2.4 | 3.9 | 8.6 | <0.001 | Statistically significant increased trend |
| 60-64 | Full Range | 1999 | 2018 | 3.8* | 2.1 | 5.5 | 4.7 | <0.001 | Statistically significant increased trend |
| 65-69 | Full Range | 1999 | 2018 | 3.8* | 2.7 | 4.8 | 7.6 | <0.001 | Statistically significant increased trend |
| 70-74 | 1 | 1999 | 2004 | 15.2* | 3.6 | 28.2 | 2.9 | <0.001 | Statistically significant increased trend |
|  | 2 | 2004 | 2007 | -4.7 | -36.0 | 41.7 | -0.3 | 0.8 | Not statistically significant |
|  | 3 | 2007 | 2018 | 3.9* | 1.4 | 6.4 | 3.4 | <0.001 | Statistically significant increased trend |
|  | Full Range | 1999 | 2018 | 3.7* | 2.2 | 5.2 | 5.1 | <0.001 | Statistically significant increased trend |
| 75+ | 1 | 1999 | 2002 | -21.8* | -38.0 | -1.3 | -2.3 | <0.001 | Statistically significant decreased trend |
|  | 2 | 2002 | 2005 | 18.1 | -26.4 | 89.6 | 0.8 | 0.5 | Not statistically significant |
|  | 3 | 2005 | 2018 | 2.3* | 0.1 | 4.5 | 2.3 | <0.001 | Statistically significant increased trend |
|  | Full Range | 1999 | 2018 | 2.1* | 0.6 | 3.7 | 2.9 | <0.001 | Statistically significant increased trend |
| **Indian/Asian** | | | | | | | | | |
| 15-19 | 1 | 1999 | 2016 | 92.3* | 92.3 | 92.3 | 4.2 x10^14^ | <0.001 | Statistically significant increased trend |
|  | 2 | 2016 | 2018 | -99.0* | -99.0 | -99.0 | -1.0 x10^13^ | <0.001 | Statistically significant decreased trend |
|  | Full Range | 1999 | 2018 | 89.3* | 50.9 | 137.6 | 5.9 | <0.001 | Statistically significant increased trend |
| 20-24 | Full Range | 1999 | 2018 | 94.6* | 58.4 | 139.0 | 6.8 | <0.001 | Statistically significant increased trend |
| 25-29 | Full Range | 1999 | 2018 | -1.9 | -6.0 | 2.3 | -1.0 | 0.4 | Not statistically significant |
| 30-34 | Full Range | 1999 | 2018 | -2.4 | -6.4 | 1.7 | -1.2 | 0.2 | Not statistically significant |
| 35-39 | 1 | 1999 | 2005 | -7.9 | -28.3 | 18.2 | -0.7 | 0.5 | Not statistically significant |
|  | 2 | 2005 | 2018 | 7.5* | 0.3 | 15.1 | 2.2 | 0.0 | Statistically significant increased trend |
|  | Full Range | 1999 | 2018 | 3.5 | -0.1 | 7.2 | 2.0 | 0.1 | Not statistically significant |
| 40-44 | Full Range | 1999 | 2018 | 0.6 | -2.7 | 4.1 | 0.4 | 0.7 | Not statistically significant |
| 45-49 | Full Range | 1999 | 2018 | -0.1 | -3.6 | 3.6 | -0.0 | 1.0 | Stable |
| 50-54 | 1 | 1999 | 2015 | 3.5* | 0.4 | 6.6 | 2.4 | <0.001 | Statistically significant increased trend |
|  | 2 | 2015 | 2018 | -26.9 | -52.4 | 12.1 | -1.6 | 0.1 | Not statistically significant |
|  | Full Range | 1999 | 2018 | 1.5 | -1.2 | 4.3 | 1.1 | 0.3 | Not statistically significant |
| 55-59 | 1 | 1999 | 2006 | 9.1 | -0.4 | 19.4 | 2.0 | 0.1 | Not statistically significant |
|  | 2 | 2006 | 2018 | -4.2* | -7.9 | -0.3 | -2.3 | <0.001 | Statistically significant decreased trend |
|  | Full Range | 1999 | 2018 | -0.3 | -2.7 | 2.1 | -0.3 | 0.8 | Stable |
| 60-64 | Full Range | 1999 | 2018 | 1.0 | -1.5 | 3.7 | 0.8 | 0.4 | Not statistically significant |
| 65-69 | Full Range | 1999 | 2018 | 2.0 | -0.5 | 4.6 | 1.6 | 0.1 | Not statistically significant |
| 70-74 | Full Range | 1999 | 2018 | 1.9 | -1.1 | 5.1 | 1.3 | 0.2 | Not statistically significant |
| 75+ | Full Range | 1999 | 2018 | 1.3 | -0.8 | 3.5 | 1.3 | 0.2 | Not statistically significant |
| **Coloured** |  |  |  |  |  |  |  |  |  |
| 15-19 | Full Range | 1999 | 2018 | 4.8 | -7.9 | 19.2 | 0.8 | 0.5 | Not statistically significant |
| 20-24 | 1 | 1999 | 2009 | 48.2 | -17.8 | 167.2 | 1.4 | 0.2 | Not statistically significant |
|  | 2 | 2009 | 2018 | -16.4* | -26.7 | -4.6 | -2.9 | <0.001 | Statistically significant decreased trend |
|  | Full Range | 1999 | 2018 | -0.2 | -7.8 | 8.0 | -0.1 | 1.0 | Stable |
| 25-29 | Full Range | 1999 | 2018 | 1.6 | -3.1 | 6.5 | 0.7 | 0.5 | Not statistically significant |
| 30-34 | Full Range | 1999 | 2018 | 1.3 | -1.3 | 3.9 | 1.1 | 0.3 | Not statistically significant |
| 35-39 | 1 | 1999 | 2002 | -15.2 | -40.2 | 20.3 | -1.1 | 0.3 | Not statistically significant |
|  | 2 | 2002 | 2005 | 23.3 | -47.1 | 187.2 | 0.6 | 0.6 | Not statistically significant |
|  | 3 | 2005 | 2008 | -17.9 | -60.4 | 70.4 | -0.6 | 0.6 | Not statistically significant |
|  | 4 | 2008 | 2018 | 6.6* | 0.8 | 12.7 | 2.6 | <0.001 | Statistically significant increased trend |
|  | Full Range | 1999 | 2018 | 1.4 | -0.8 | 3.6 | 1.3 | 0.2 | Not statistically significant |
| 40-44 | Full Range | 1999 | 2018 | 1.9 | -0.3 | 4.2 | 1.8 | 0.1 | Not statistically significant |
| 45-49 | 1 | 1999 | 2002 | -13.3 | -27.1 | 3.1 | -1.9 | 0.1 | Not statistically significant |
|  | 2 | 2002 | 2005 | 15.0 | -19.5 | 64.3 | 0.9 | 0.4 | Not statistically significant |
|  | 3 | 2005 | 2008 | -5.6 | -34.3 | 35.5 | -0.4 | 0.7 | Not statistically significant |
|  | 4 | 2008 | 2018 | 4.2* | 1.7 | 6.8 | 3.8 | <0.001 | Statistically significant increased trend |
|  | Full Range | 1999 | 2018 | 2.2* | 0.9 | 3.5 | 3.5 | <0.001 | Statistically significant increased trend |
| 50-54 | 1 | 1999 | 2006 | 4.5 | -2.4 | 12.0 | 1.4 | 0.2 | Not statistically significant |
|  | 2 | 2006 | 2018 | 0.1 | -2.8 | 3.1 | 0.1 | 0.9 | Not statistically significant |
|  | Full Range | 1999 | 2018 | 1.4 | -0.0 | 2.8 | 2.0 | 0.1 | Not statistically significant |
| 55-59 | Full Range | 1999 | 2018 | 2.4* | 1.3 | 3.5 | 4.6 | <0.001 | Statistically significant increased trend |
| 60-64 | Full Range | 1999 | 2018 | 3.6* | 1.3 | 5.9 | 3.4 | <0.001 | Statistically significant increased trend |
| 65-69 | 1 | 1999 | 2001 | 19.1 | -34.4 | 116.3 | 0.6 | 0.5 | Not statistically significant |
|  | 2 | 2001 | 2018 | 2.2* | 0.5 | 4.0 | 2.7 | <0.001 | Statistically significant increased trend |
|  | Full Range | 1999 | 2018 | 2.7* | 1.2 | 4.2 | 3.8 | <0.001 | Statistically significant increased trend |
| 70-74 | 1 | 1999 | 2006 | 0.9 | -3.4 | 5.3 | 0.4 | 0.7 | Not statistically significant |
|  | 2 | 2006 | 2018 | 4.0* | 2.3 | 5.7 | 5.1 | <0.001 | Statistically significant increased trend |
|  | Full Range | 1999 | 2018 | 3.1* | 1.9 | 4.2 | 5.6 | <0.001 | Statistically significant increased trend |
| 75+ | Full Range | 1999 | 2018 | 3.5* | 1.7 | 5.2 | 4.2 | <0.001 | Statistically significant increased trend |
| **White** |  |  |  |  |  |  |  |  |  |
| 15-19 | 1 | 1999 | 2009 | 150.1* | 150.1 | 150.1 | 9.8 x^14^ | <0.001 | Statistically significant increased trend |
|  | 2 | 2009 | 2018 | -63.7* | -63.7 | -63.7 | -9.2x10^14^ | <0.001 | Statistically significant decreased trend |
|  | Full Range | 1999 | 2018 | 9.8 | -35.3 | 86.3 | 0.4 | 0.7 | Not statistically significant |
| 20-24 | Full Range | 1999 | 2018 | -4.6* | -8.6 | -0.4 | -2.3 | <0.001 | Statistically significant decreased trend |
| 25-29 | Full Range | 1999 | 2018 | -2.1 | -5.9 | 2.0 | -1.1 | 0.3 | Not statistically significant |
| 30-34 | Full Range | 1999 | 2018 | 5.2* | 1.6 | 8.9 | 3.0 | <0.001 | Statistically significant increased trend |
| 35-39 | Full Range | 1999 | 2018 | 3.5* | 1.8 | 5.2 | 4.3 | <0.001 | Statistically significant increased trend |
| 40-44 | Full Range | 1999 | 2018 | 3.0* | 1.2 | 4.7 | 3.6 | <0.001 | Statistically significant increased trend |
| 45-49 | 1 | 1999 | 2008 | -4.1* | -6.9 | -1.3 | -3.1 | <0.001 | Statistically significant decreased trend |
|  | 2 | 2008 | 2018 | 5.6* | 3.1 | 8.2 | 4.9 | <0.001 | Statistically significant increased trend |
|  | Full Range | 1999 | 2018 | 1.0 | -0.4 | 2.4 | 1.5 | 0.1 | Not statistically significant |
| 50-54 | 1 | 1999 | 2005 | -6.9* | -12.8 | -0.5 | -2.3 | <0.001 | Statistically significant decreased trend |
|  | 2 | 2005 | 2018 | 0.3 | -2.0 | 2.6 | 0.3 | 0.8 | Not statistically significant |
|  | Full Range | 1999 | 2018 | -1.7* | -3.0 | -0.5 | -2.9 | <0.001 | Statistically significant decreased trend |
| 55-59 | Full Range | 1999 | 2018 | -0.03 | -0.9 | 0.9 | -0.1 | 0.9 | Stable |
| 60-64 | 1 | 1999 | 2016 | 0.9* | 0.1 | 1.8 | 2.3 | 0.0 | Statistically significant increased trend |
|  | 2 | 2016 | 2018 | -9.1 | -29.7 | 17.5 | -0.8 | 0.4 | Not statistically significant |
|  | Full Range | 1999 | 2018 | 0.5 | -0.3 | 1.4 | 1.4 | 0.2 | Stable |
| 65-69 | Full Range | 1999 | 2018 | 0.6 | -0.7 | 1.8 | 1.0 | 0.3 | Not statistically significant |
| 70-74 | 1 | 1999 | 2001 | 18.0 | -19.6 | 73.0 | 0.9 | 0.4 | Not statistically significant |
|  | 2 | 2001 | 2018 | 0.3 | -0.9 | 1.5 | 0.5 | 0.7 | Stable |
|  | Full Range | 1999 | 2018 | 0.8 | -0.2 | 1.8 | 1.7 | 0.1 | Not statistically significant |
| 75+ | Full Range | 1999 | 2018 | 0.9* | 0.1 | 1.7 | 2.5 | <0.001 | Statistically significant increased trend |

**Supplementary Table 4. Age Period Cohort effect estimates of trends in Breast cancer mortality in South Africa, 1999-2018.**

| **Factor** | **Overall** |  |  |  |  |  |
| --- | --- | --- | --- | --- | --- | --- |
|  | **Longitudinal RR(adjusted for period effect)** | **95%CI** | **Cross sectional RR (Adjusted for cohort effect)** | **95%CI** | **Local drift(%)** | **95%CI** |
| Age |  |  |  |  |  |  |
| 15-19 | 0.04 | 0.02-0.09 | 0.06 | 0.03-0.14 | -0.23 | -9.86 to 10.42 |
| 20-24 | 0.17 | 0.11-0.25 | 0.27 | 0.19-0.38 | -0.24 | -4.50 to 4.21 |
| 25-29 | 0.86 | 0.69-1.07 | 1.28 | 1.05-1.57 | 1.18 | -0.83 to 3.23 |
| 30-34 | 2.86 | 2.47-3.30 | 3.95 | 3.42-4.57 | 2.44 | 1.25-3.65 |
| 35-39 | 7.21 | 6.47-8.03 | 9.26 | 8.22-10.44 | 2.26 | 1.39-3.15 |
| 40-44 | 13.67 | 12.59-14.83 | 16.32 | 14.79-18.01 | 1.53 | 0.83-2.24 |
| 45-49 | 21.19 | 19.80-22.67 | 23.52 | 21.68-25.51 | 1.52 | 0.89-2.16 |
| 50-54 | 29.34 | 27.57-31.22 | 30.27 | 28.26-32.43 | 1.12 | 0.53-1.72 |
| 55-59 | 39.68 | 37.22-42.30 | 38.05 | 35.73-40.52 | 1.02 | 0.44-1.61 |
| 60-64 | 46.67 | 42.95-50.71 | 41.60 | 38.90-44.48 | 1.40 | 0.81-2.00 |
| 65-69 | 59.66 | 54.34-65.50 | 49.43 | 45.64-53.53 | 1.67 | 1.05-2.30 |
| 70-74 | 77.20 | 69.48-85.77 | 59.45 | 53.76-65.74 | 1.54 | 0.92-2.16 |
| 75+ | 150.63 | 135.28-167.73 | 107.81 | 95.95-121.15 | 2.81 | 2.17-3.45 |
| **Net Drift (%)** |  |  |  |  | 1.47 | 0.91 – 2.04 |
| **Period** | Rate Ratio |  |  |  |  |  |
| 1999-2003 | 0.82 | 0.77-0.87 |  |  |  |  |
| 2004-2008 | 1.00 | 1.00-1.00 |  |  |  |  |
| 2009-2013 | 1.09 | 0.95-1.06 |  |  |  |  |
| 2014- 2018 | 1.04 | 0.98-1.11 |  |  |  |  |
| **Cohort** |  |  |  |  |  |  |
| 1924-1928 | 0.50 | 0.43-0.57 |  |  |  |  |
| 1929-1933 | 0.67 | 0.59-0.76 |  |  |  |  |
| 1934-1938 | 0.72 | 0.65-0.81 |  |  |  |  |
| 1939-1943 | 0.77 | 0.69-0.85 |  |  |  |  |
| 1944-1948 | 0.85 | 0.78-0.93 |  |  |  |  |
| 1949-1953 | 0.92 | 0.85-1.00 |  |  |  |  |
| 1954-1959 | 0.94 | 0.88-1.01 |  |  |  |  |
| 1959-1963 | 1.00 | 1.00-1.00 |  |  |  |  |
| 1964-1968 | 1.09 | 1.01-1.17 |  |  |  |  |
| 1969-1973 | 1.17 | 1.08-1.28 |  |  |  |  |
| 1974-1978 | 1.26 | 1.13-1.40 |  |  |  |  |
| 1979-1983 | 1.55 | 1.34-1.78 |  |  |  |  |
| 1984-1988 | 1.64 | 1.35-1.99 |  |  |  |  |
| 1989-1993 | 1.50 | 1.08-2.09 |  |  |  |  |
| 1994 -1998 | 1.53 | 0.75-3.14 |  |  |  |  |
| 1999-2003 | 1.57 | 0.30-8.06 |  |  |  |  |

**Supplementary Table 5. Age Period Cohort effect estimates of ethnic trends in Breast cancer mortality by ethnic groups in South Africa, 1999-2018.**

| **Factor** | **BLACK** | | | **WHITE** | | | **INDIAN/ASIAN** | | | **COLOURED** | | |
| --- | --- | --- | --- | --- | --- | --- | --- | --- | --- | --- | --- | --- |
|  | **Longitudinal RR(adjusted for period effect(95%CI)** | **Cross sectional RR (Adjusted for cohort effect(95%CI)** | **Local drift(%)**  **(95%CI)** | **Longitudinal RR(adjusted for period effect(95%CI)** | **Cross sectional RR (Adjusted for cohort effect(95%CI)** | **Local drift(%)**  **(95%CI)** | **Longitudinal RR(adjusted for period effect(95%CI)** | **Cross sectional RR (Adjusted for cohort effect(95%CI)** | **Local drift(%)**  **(95%CI)** | **Longitudinal RR(adjusted for period effect(95%CI)** | **Cross sectional RR (Adjusted for cohort effect(95%CI)** | **Local drift(%)**  **(95%CI)** |
| **Age** |  |  |  |  |  |  |  |  |  |  |  |  |
| 15-19 | 0.018  (0.008-0.042) | 0.065  (0.032-0.135) | -1.448  (-10.694to8.755) | 0.042  (0.002-0.730) | 0.062  (0.005-0.741) | -9.789  (-43.814to44.841) | 0.030  (0.001-1.664) | 0.037  (0.001-1.225) | 24.338  (-6990to66.217) | 0.033  (0.003-0.315) | 0.069  (0.010-0.489) | 3.212  (-15.063to25.421) |
| 20-24 | 0.076  (0.050-0.115) | 0.217  (0.153-0.309) | 2.014  (-2.163to6.369) | 0.092  (0.026-0.329) | 0.129  (0.038-0.439) | -0.110  (-13.472to15.315) | 0.097  (0.003-3.102) | 0.116  (0.006-2.205) | 9.101  (-13.143to37.041) | 0.104  (0.037-0.289) | 0.192  (0.080-0.457) | 1.587  (-8.087to12.279) |
| 25-29 | 0.40  (0.32-0.51) | 0.92  (0.75-1.13) | 3.58  (1.56to5.65) | 0.57  (0.34-0.98) | 0.77  (0.40-1.51) | -1.09  (-7.57to5.85) | 1.22  (0.49-3.06) | 1.42  (0.59-3.41) | -5.91  (-17.10to6.79) | 1.03  (0.72-1.48) | 1.69  (1.17-2.45) | 1.51  (-2.10to5.24) |
| 30-34 | 1.46  (1.24-1.72) | 2.68  (2.31-3.11) | 4.24  (2.96to5.54) | 2.86  (2.15-3.82) | 3.70  (2.28-6.01) | 4.59  (1.99to7.26) | 2.45  (1.32-4.52) | 2.77  (1.42-5.41) | 2.00  (-3.13to7.40) | 2.79  (2.20-3.54) | 4.05  (3.03-5.41) | 2.93  (0.88to5.02) |
| 35-39 | 3.99  (3.53-4.51) | 5.87  (5.18-6.64) | 4.18  (3.22to5.15) | 6.57  (5.39-8.02) | 8.16  (5.58-11.95) | 3.66  (1.89to5.47) | 8.11  (5.49-11.98) | 8.94  (5.41-14.77) | 1.03  (-2.18to4.35) | 6.55  (5.51-7.80) | 8.42  (6.66-10.64) | 2.16  (0.72to3.62) |
| 40-44 | 8.61  (7.82-9.49) | 10.13  (9.10-11.28) | 4.74  (3.92to5.56) | 14.98  (13.12-17.11) | 17.85  (13.45-23.69) | 2.82  (1.58to4.07) | 12.93  (9.76-17.14) | 13.88  (9.26-20.82) | -1.10  (-3.60to1.45) | 14.40  (12.68-16.34) | 16.38  (13.63-19.68) | 1.76  (0.65to2.88) |
| 45-49 | 14.63  (13.49-15.88) | 13.78  (12.53-15.15) | 5.13  (4.34to5.91) | 23.78  (21.37-26.47) | 27.19  (22.40-33.00) | 0.98  (-0.01to1.97) | 21.44  (17.42-26.40) | 22.41  (16.58-30.30) | -1.79  (-3.83to0.28) | 24.34  (21.92-27.02) | 24.52  (21.29-28.25) | 1.86  (0.91to2.82) |
| 50-54 | 22.13  (20.52-23.85) | 16.68  (15.29-18.19) | 5.32  (4.53to6.11) | 35.21  (32.15-38.56) | 38.63  (34.45-43.31) | -0.31  (-1.13to0.52) | 32.60  (27.17-39.11) | 33.17  (26.73-41.17) | -1.25  (-2.93to0.47) | 37.33  (34.04-40.93) | 33.31  (29.88-37.14) | 1.96  (1.08to2.86) |
| 55-59 | 34.22  (31.67-36.98) | 20.66  (18.99-22.47) | 5.40  (4.58to6.21) | 45.12  (41.28-49.31) | 47.50  (43.49-51.87) | -0.47  (-1.20to0.27) | 45.25  (37.63-54.41) | 44.84  (37.76-53.25) | -0.82  (-2.38to0.76) | 57.34  (52.53-62.60) | 45.33  (41.16-49.92) | 2.50  (1.61to3.40) |
| 60-64 | 43.98  (39.51-48.95) | 21.25  (19.45-23.22) | 5.18  (4.32to6.04) | 56.56  (50.68-63.12) | 57.13  (49.38-66.11) | 0.21  (-0.49to0.92) | 48.99  (38.89-61.70) | 47.26  (38.61-57.85) | 0.21  (-1.30to1.75) | 75.28  (66.75-84.90) | 52.71  (47.08-59.00) | 3.41  (2.47to4.36) |
| 65-69 | 63.98  (56.53-72.40) | 24.75  (22.44-27.31) | 5.34  (4.42to6.27) | 73.07  (64.94-82.21) | 70.83  (56.14-89.36) | 0.36  (-0.32to1.04) | 63.76  (49.53-82.07) | 59.90  (45.44-78.95) | 1.31  (-0.34to3.00) | 109.02  (94.88-125.26) | 67.61  (58.36-78.32) | 3.63  (2.64to4.64) |
| 70-74 | 94.01  (81.49-108.44) | 29.12  (25.90-32.75) | 5.40  (4.44to6.37) | 92.30  (81.15-104.99) | 85.86-(61.95-118.99) | 0.38  (-0.25to1.03) | 73.39  (55.15-97.67) | 67.13  (45.99-98.00) | 0.17  (-1.55to1.92) | 162.12  (138.29-190.04) | 89.04  (73.32-108.13) | 3.33  (2.30to4.37) |
| 75+ | 214.30  (184.84—248.46) | 53.16(46.84-60.34) | 5.60  (4.56-6.66) | 179.03  (157.22-203.87) | 159.81  (105.06-243.07) | 1.37  (0.79to1.95) | 116.83  (86.81-157.25) | 104.07  (64.91-166.83) | 0.09  (-2.04to2.27) | 283.04  (239.51-334.49) | 137.70  (108.58-174.63) | 3.86  (2.66to5.07) |
| **Net Drift (%)** |  |  | 4.55  (3.94to5.16) |  |  | 0.83  (-1.13to2.82) |  |  | 0.53  (-1.79to2.91) |  |  | 2.46  (1.30t03.62) |
| **Period** | Rate Ratio  (95%CI) |  |  | Rate Ratio  (95%CI) |  |  | Rate Ratio  (95%CI) |  |  | Rate Ratio  (95%CI) |  |  |
| 1999-2003 | 0.78  (0.73-0.84) |  |  | 1.00  (0.89-1.12) |  |  | 1.02  (0.85-1.24) |  |  | 0.81  (0.74-0.90) |  |  |
| 2004-2008 | 1.00  (1.00-1.00) |  |  | 1.00  (1.00-1.00) |  |  | 1.00  (1.00-1.00) |  |  | 1.00  (1.00-1.00) |  |  |
| 2009-2013 | 1.09  (1.02-1.16) |  |  | 1.07  (0.95-1.19) |  |  | 1.05  (0.89-1.25) |  |  | 1.02  (0.94-11.12) |  |  |
| 2014- 2018 | 1.59  (1.48-1.71) |  |  | 1.12  (0.92-1.37) |  |  | 1.10  (0.86-1.41) |  |  | 1.21  (1.07-1.36) |  |  |
| **Cohort** | Rate Ratio  (95%CI) |  |  | Rate Ratio  (95%CI) |  |  | Rate Ratio  (95%CI) |  |  | Rate Ratio  (95%CI) |  |  |
| 1924-1928 | 0.16  (0.13-0.19 |  |  | 0.80  (0.69-0.93) |  |  | 1.00  (0.65-1.54) |  |  | 0.31  (0.25-0.40) |  |  |
| 1929-1933 | 0.21  (0.17-0.25) |  |  | 0.97  (0.84-1.12) |  |  | 1.14  (0.81-1.61) |  |  | 0.42  (0.35-0.52) |  |  |
| 1934-1938 | 0.27  (0.23-0.31) |  |  | 1.05  (0.91-1.20) |  |  | 0.96  (0.70-1.31) |  |  | 0.48  (0.40-0.57) |  |  |
| 1939-1943 | 0.36  (0.31-0.41) |  |  | 0.98  (0.86-1.11) |  |  | 1.08  (0.82-1.42) |  |  | 0.56  (0.48-0.66) |  |  |
| 1944-1948 | 0.45  (0.40-0.51) |  |  | 1.05  (0.94-1.18) |  |  | 1.13  (0.88-1.44) |  |  | 0.69  (0.61-0.79) |  |  |
| 1949-1953 | 0.59  (0.54-0.66) |  |  | 1.08  (0.98-1.20) |  |  | 1.17  (0.94-1.46) |  |  | 0.81  (0.73-0.91) |  |  |
| 1954-1959 | 0.75  (0.69-0.82) |  |  | 1.00  (0.91-1.11) |  |  | 1.10  (0.90-1.35) |  |  | 0.94  (0.84-1.04) |  |  |
| 1959-1963 | 1.00  (1.00-1.00) |  |  | 1.00  (1.00-1.00) |  |  | 1.00  (1.00-1.00) |  |  | 1.00  (1.00-1.00) |  |  |
| 1964-1968 | 1.28  (1.17-1.40) |  |  | 1.03  (0.92-1.16) |  |  | 0.98  (0.78-1.24) |  |  | 1.10  (0.99-1.23) |  |  |
| 1969-1973 | 1.60  (1.44-1.77) |  |  | 1.17  (1.01-1.35) |  |  | 0.82  (0.61-1.11) |  |  | 1.23  (1.08-1.41) |  |  |
| 1974-1978 | 2.01  (1.78-2.27) |  |  | 1.52  (1.26-1.84) |  |  | 0.88  (0.59-1.31) |  |  | 1.29  (1.09-1.53) |  |  |
| 1979-1983 | 2.35  (2.01-2.74) |  |  | 1.72  (1.30-2.28) |  |  | 1.14  (0.67-1.92) |  |  | 1.55  (1.22-1.96) |  |  |
| 1984-1988 | 3.03  (2.46-3.74) |  |  | 2.38  (1.58-3.57) |  |  | 1.05  (0.45-2.44) |  |  | 1.87  (1.34-2.61) |  |  |
| 1989-1993 | 3.32  (2.38-4.63) |  |  | 1.14  (0.37-3.48) |  |  | 0.33  (0.04-2.65) |  |  | 1.55  (0.86-2.81) |  |  |
| 1994 -1998 | 3.18  (1.59-6.34) |  |  | 2.16  (0.20-22.84) |  |  | 7.15  (0.19-273.49) |  |  | 2.14  (0.42-11.07) |  |  |
| 1999-2003 | 2.41  (0.49-11.91) |  |  | 0.34  (0.00-731.87) |  |  | 14.18  (0.16-1271.55) |  |  | 2.85  (0.14-56.98) |  |  |
|  |  |  |  |  |  |  |  |  |  |  |  |  |
