# Supplementary material for: Population-based temporal trends and ethnic disparity in breast cancer mortality in South Africa (1999-2018): Joinpoint and age–period–cohort regression analyses
Source: Front Oncol. 2023 Feb 3;13:1056609. doi: 10.3389/fonc.2023.1056609 (PMC9935608; doi:10.3389/fonc.2023.1056609)
Supplement: Supplementary file 2 [file DataSheet_1.pdf]

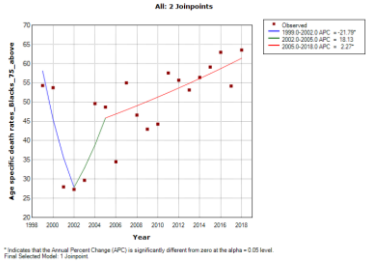
All: 2 Joinpoints
Observed
1999.0-2002.0 APC = -21.79*
2002.0-2005.0 APC = 18.13
2005.0-2018.0 APC = 2.27*
Age specific death rates _Blacks_75_above
Year
* Indicates that the Annual Percent Change (APC) is significantly different from zero at the alpha = 0.05 level.
Final Selected Model: 1 Joinpoint.

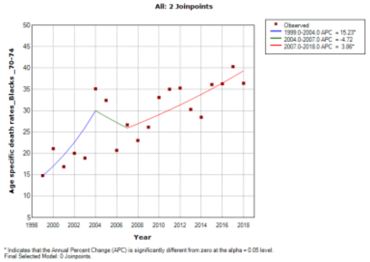
All: 2 Joinpoints
Observed
1999.0-2004.0 APC = 15.23*
2004.0-2007.0 APC = -4.72
2007.0-2018.0 APC = 3.86*
Age specific death rates _Blacks _70-74
Year
* Indicates that the Annual Percent Change (APC) is significantly different from zero at the alpha = 0.05 level.
Final Selected Model: 0 Joinpoints.

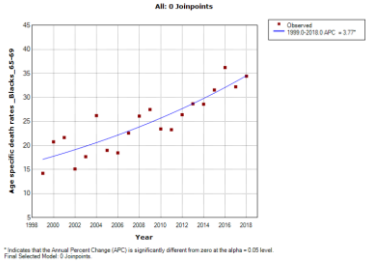
All: 0 Joinpoints
Observed
1999.0-2018.0 APC = 3.77*
Age specific death rates _Blacks_65-69
Year
* Indicates that the Annual Percent Change (APC) is significantly different from zero at the alpha = 0.05 level.
Final Selected Model: 0 Joinpoints.

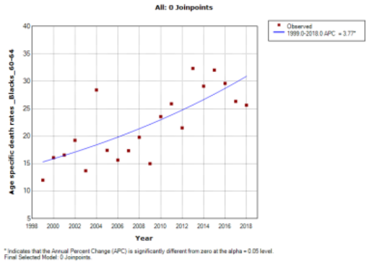
All: 0 Joinpoints
Observed
1999.0-2018.0 APC = 3.77*
Age specific death rates _Blacks_60-64
Year
* Indicates that the Annual Percent Change (APC) is significantly different from zero at the alpha = 0.05 level.
Final Selected Model: 0 Joinpoints.

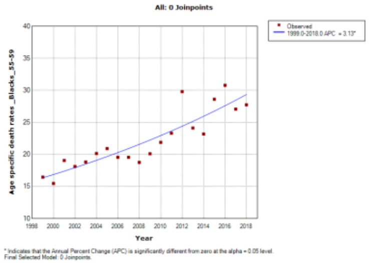
All: 0 Joinpoints
Observed
1999.0-2018.0 APC = 3.13*
Age specific death rates _Blacks_55-59
Year
* Indicates that the Annual Percent Change (APC) is significantly different from zero at the alpha = 0.05 level.
Final Selected Model: 0 Joinpoints.

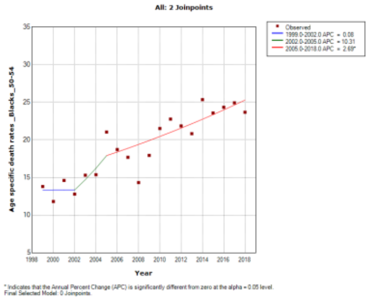
All: 2 Joinpoints
Observed
1999.0-2002.0 APC = 0.08
2002.0-2005.0 APC = 10.31
2005.0-2018.0 APC = 2.69*
Age specific death rates _Blacks_50-54
Year
* Indicates that the Annual Percent Change (APC) is significantly different from zero at the alpha = 0.05 level.
Final Selected Model: 0 Joinpoints.

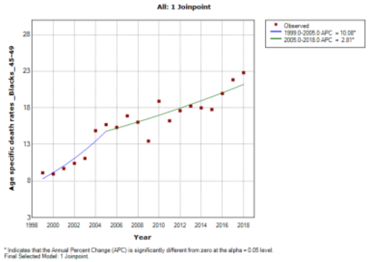
All: 1 Joinpoint
Observed
1999.0-2005.0 APC = 10.08*
2005.0-2018.0 APC = 2.81*
Age specific death rates _Blacks_45-49
Year
* Indicates that the Annual Percent Change (APC) is significantly different from zero at the alpha = 0.05 level.
Final Selected Model: 1 Joinpoint.

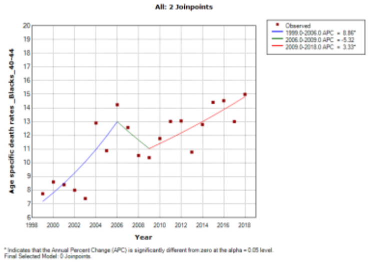
All: 2 Joinpoints
Observed
1999.0-2006.0 APC = 8.86*
2006.0-2009.0 APC = -5.32
2009.0-2018.0 APC = 3.33*
Age specific death rates _Blacks_40-44
Year
* Indicates that the Annual Percent Change (APC) is significantly different from zero at the alpha = 0.05 level.
Final Selected Model: 0 Joinpoints.

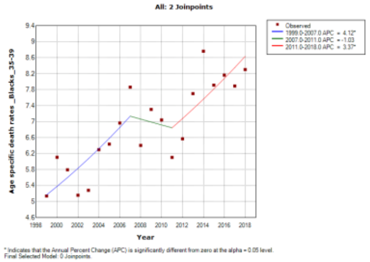
All: 2 Joinpoints
Observed
1999.0-2007.0 APC = 4.12*
2007.0-2011.0 APC = -1.03
2011.0-2018.0 APC = 3.37*
Age specific death rates _Blacks_35-39
Year
* Indicates that the Annual Percent Change (APC) is significantly different from zero at the alpha = 0.05 level.
Final Selected Model: 0 Joinpoints.

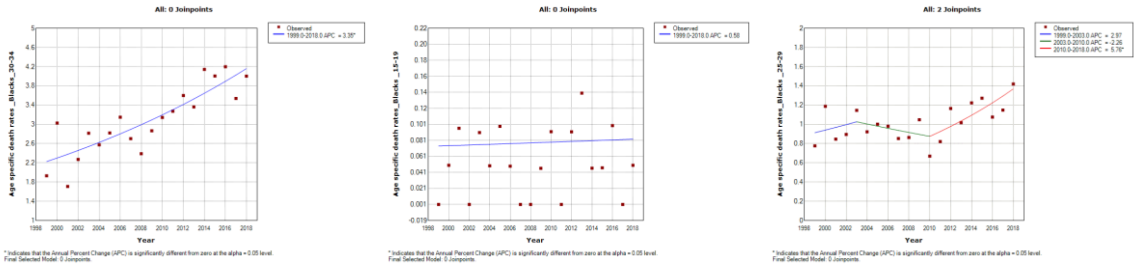

Supplementary Figure 1. Join point regression of the age specific death rates of breast cancer among Black South African

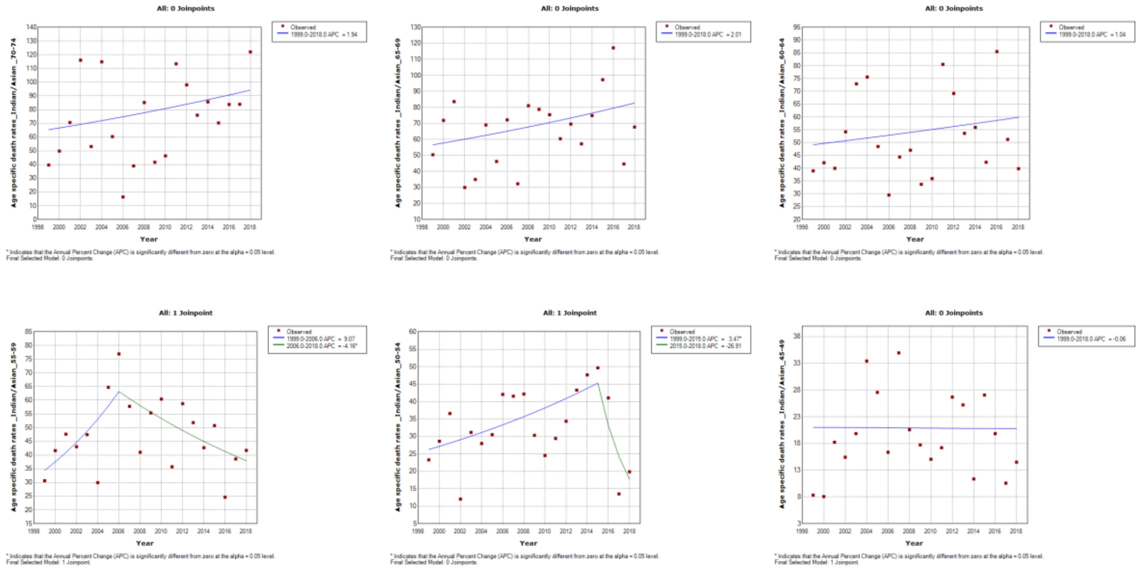

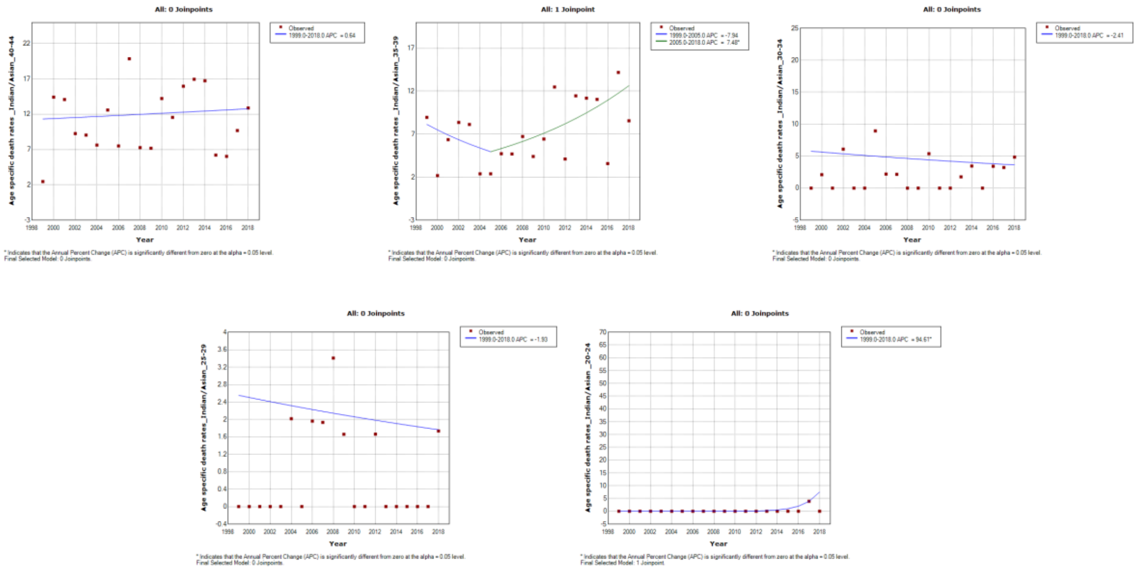

Supplementary Figure 2. Join point regression of the age specific death rates of breast cancer among Indian/Asians in South Africa (1999 – 2018)

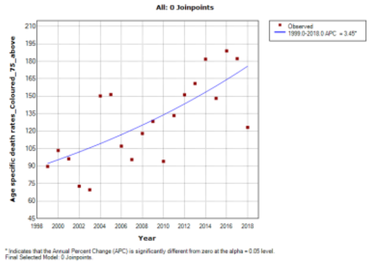
All: 0 Joinpoints
Age specific death rates_Coloured_75_above
Year
Observed
1999.0-2018.0 APC = 3.45*
* Indicates that the Annual Percent Change (APC) is significantly different from zero at the alpha = 0.05 level.
Final Selected Model: 0 Joinpoints.

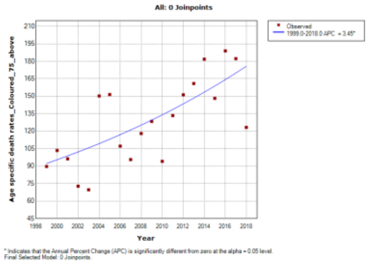
All: 0 Joinpoints
Age specific death rates_Coloured_75_above
Year
Observed
1999.0-2018.0 APC = 3.45*
* Indicates that the Annual Percent Change (APC) is significantly different from zero at the alpha = 0.05 level.
Final Selected Model: 0 Joinpoints.

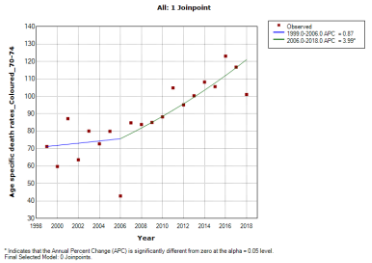
All: 1 Joinpoint
Age specific death rates_Coloured_70-74
Year
Observed
1999.0-2006.0 APC = 0.87
2006.0-2018.0 APC = 3.99*
* Indicates that the Annual Percent Change (APC) is significantly different from zero at the alpha = 0.05 level.
Final Selected Model: 0 Joinpoints.

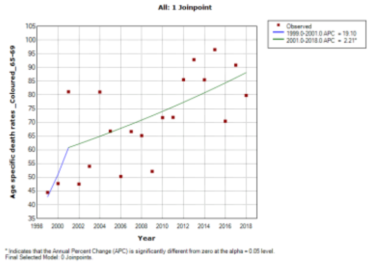
All: 1 Joinpoint
Age specific death rates _Coloured_65-69
Year
Observed
1999.0-2001.0 APC = 19.10
2001.0-2018.0 APC = 2.21*
* Indicates that the Annual Percent Change (APC) is significantly different from zero at the alpha = 0.05 level.
Final Selected Model: 0 Joinpoints.

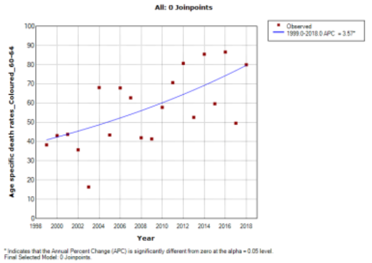
All: 0 Joinpoints
Age specific death rates_Coloured_60-64
Year
Observed
1999.0-2018.0 APC = 3.57*
* Indicates that the Annual Percent Change (APC) is significantly different from zero at the alpha = 0.05 level.
Final Selected Model: 0 Joinpoints.

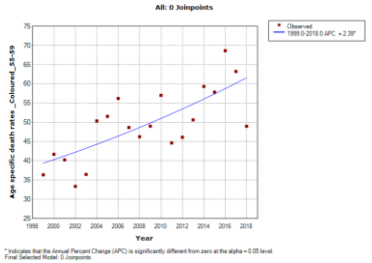
All: 0 Joinpoints
Age specific death rates _Coloured_55-59
Year
Observed
1999.0-2018.0 APC = 2.39*
* Indicates that the Annual Percent Change (APC) is significantly different from zero at the alpha = 0.05 level.
Final Selected Model: 0 Joinpoints.

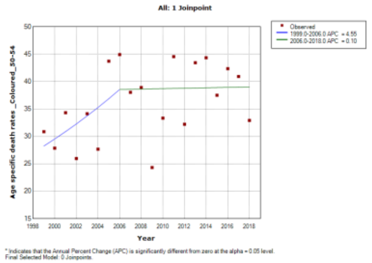
All: 1 Joinpoint
Age specific death rates _Coloured_50-54
Year
Observed
1999.0-2006.0 APC = 4.55
2006.0-2018.0 APC = 0.10
* Indicates that the Annual Percent Change (APC) is significantly different from zero at the alpha = 0.05 level.
Final Selected Model: 0 Joinpoints.

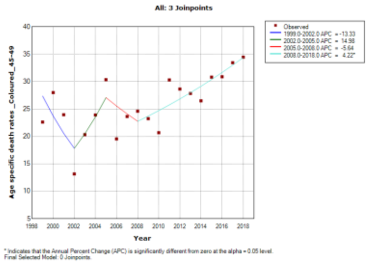
All: 3 Joinpoints
Age specific death rates _Coloured_45-49
Year
Observed
1999.0-2002.0 APC = -13.33
2002.0-2005.0 APC = 14.98
2005.0-2008.0 APC = -5.64
2008.0-2018.0 APC = 4.22*
* Indicates that the Annual Percent Change (APC) is significantly different from zero at the alpha = 0.05 level.
Final Selected Model: 0 Joinpoints.

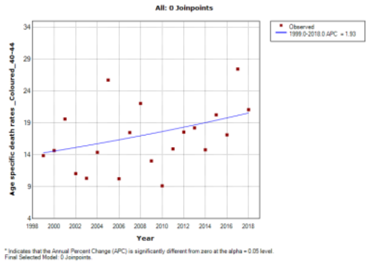
All: 0 Joinpoints
Age specific death rates _Coloured_40-44
Year
Observed
1999.0-2018.0 APC = 1.93
* Indicates that the Annual Percent Change (APC) is significantly different from zero at the alpha = 0.05 level.
Final Selected Model: 0 Joinpoints.

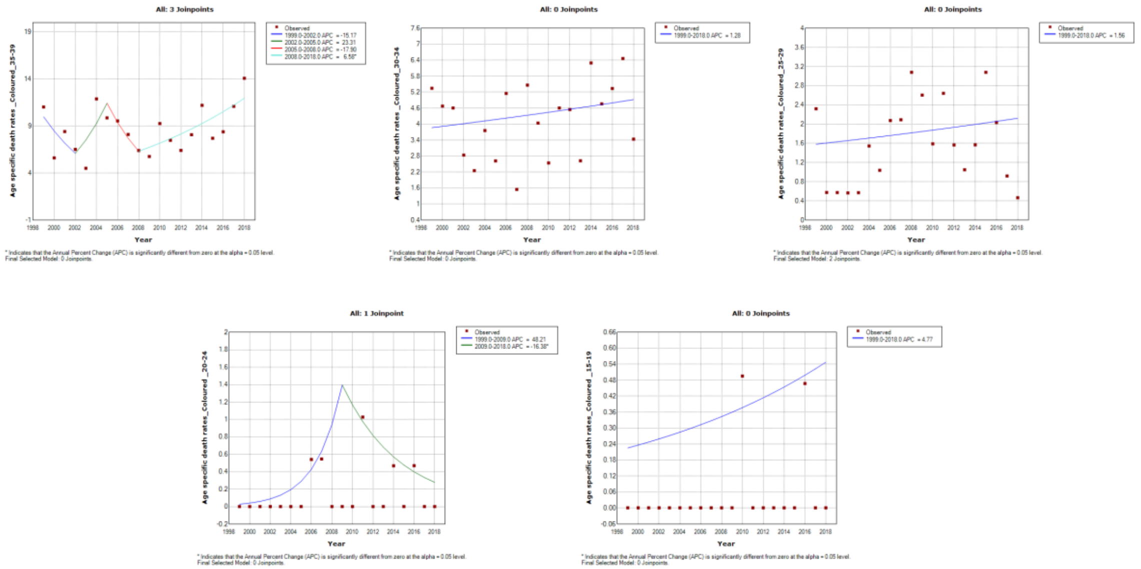

Supplementary Figure 3. Join point regression of the age specific death rates of breast cancer among Coloured in South Africa (1999 – 2018)

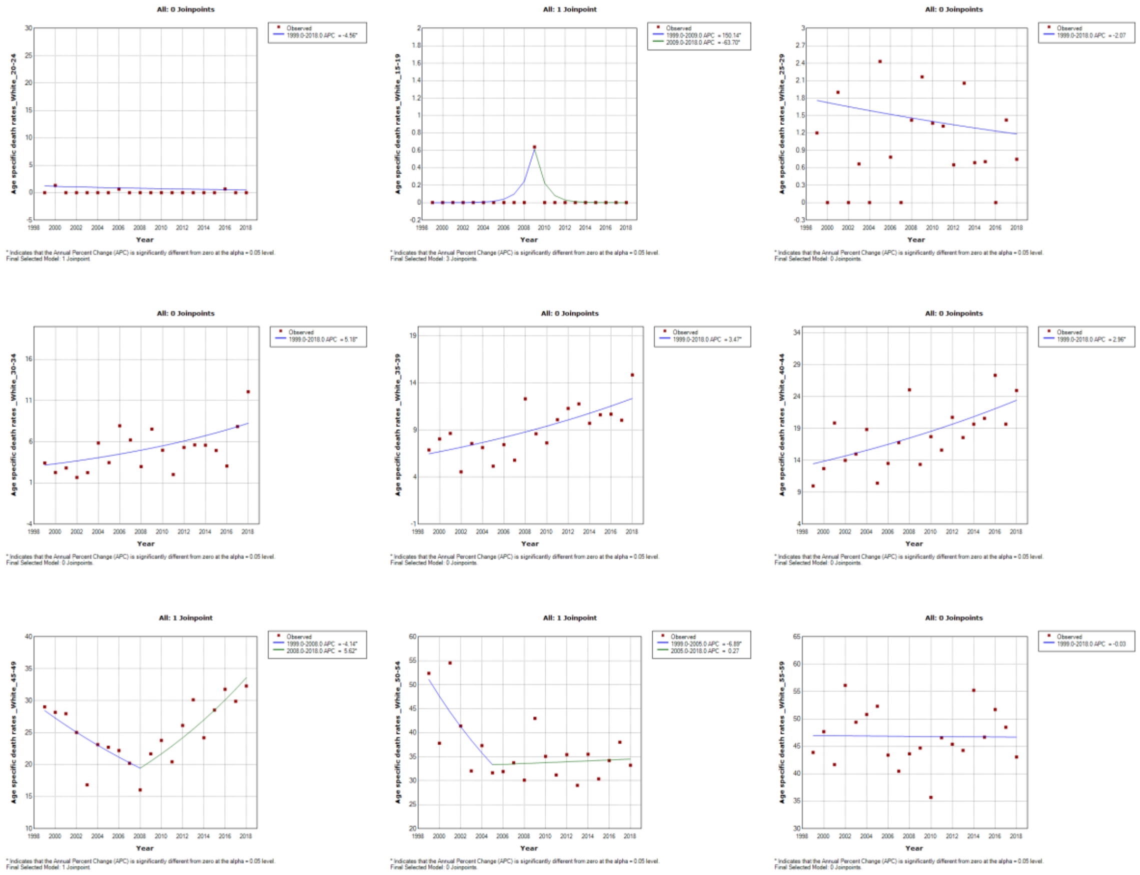
All: 0 Joinpoints
Age specific death rates _White_20-24
Observed
1999.0-2018.0 APC = -4.56*
Year
* Indicates that the Annual Percent Change (APC) is significantly different from zero at the alpha = 0.05 level.
Final Selected Model: 1 Joinpoint.
All: 1 Joinpoint
Age specific death rates _White_15-19
Observed
1999.0-2009.0 APC = 150.14*
2009.0-2018.0 APC = -63.70*
Year
* Indicates that the Annual Percent Change (APC) is significantly different from zero at the alpha = 0.05 level.
Final Selected Model: 3 Joinpoints.
All: 0 Joinpoints
Age specific death rates _White_25-29
Observed
1999.0-2018.0 APC = -2.07
Year
* Indicates that the Annual Percent Change (APC) is significantly different from zero at the alpha = 0.05 level.
Final Selected Model: 0 Joinpoints.
All: 0 Joinpoints
Age specific death rates _White_30-34
Observed
1999.0-2018.0 APC = 5.18*
Year
* Indicates that the Annual Percent Change (APC) is significantly different from zero at the alpha = 0.05 level.
Final Selected Model: 0 Joinpoints.
All: 0 Joinpoints
Age specific death rates _White_35-39
Observed
1999.0-2018.0 APC = 3.47*
Year
* Indicates that the Annual Percent Change (APC) is significantly different from zero at the alpha = 0.05 level.
Final Selected Model: 0 Joinpoints.
All: 0 Joinpoints
Age specific death rates _White_40-44
Observed
1999.0-2018.0 APC = 2.96*
Year
* Indicates that the Annual Percent Change (APC) is significantly different from zero at the alpha = 0.05 level.
Final Selected Model: 0 Joinpoints.
All: 1 Joinpoint
Age specific death rates _White_45-49
Observed
1999.0-2008.0 APC = -4.14*
2008.0-2018.0 APC = 5.62*
Year
* Indicates that the Annual Percent Change (APC) is significantly different from zero at the alpha = 0.05 level.
Final Selected Model: 1 Joinpoint.
All: 1 Joinpoint
Age specific death rates _White_50-54
Observed
1999.0-2005.0 APC = -6.89*
2005.0-2018.0 APC = 0.27
Year
* Indicates that the Annual Percent Change (APC) is significantly different from zero at the alpha = 0.05 level.
Final Selected Model: 0 Joinpoints.
All: 0 Joinpoints
Age specific death rates _White_55-59
Observed
1999.0-2018.0 APC = -0.03
Year
* Indicates that the Annual Percent Change (APC) is significantly different from zero at the alpha = 0.05 level.
Final Selected Model: 0 Joinpoints.

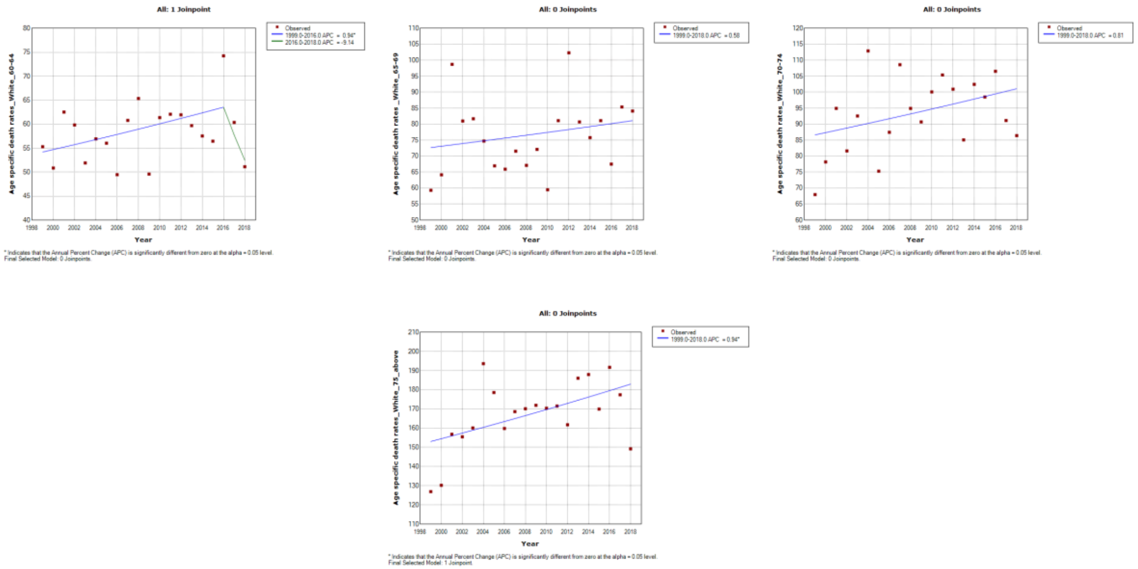

Supplementary Figure 4. Join point regression of the age specific death rates of breast cancer among Whites in South Africa (1999 – 2018)

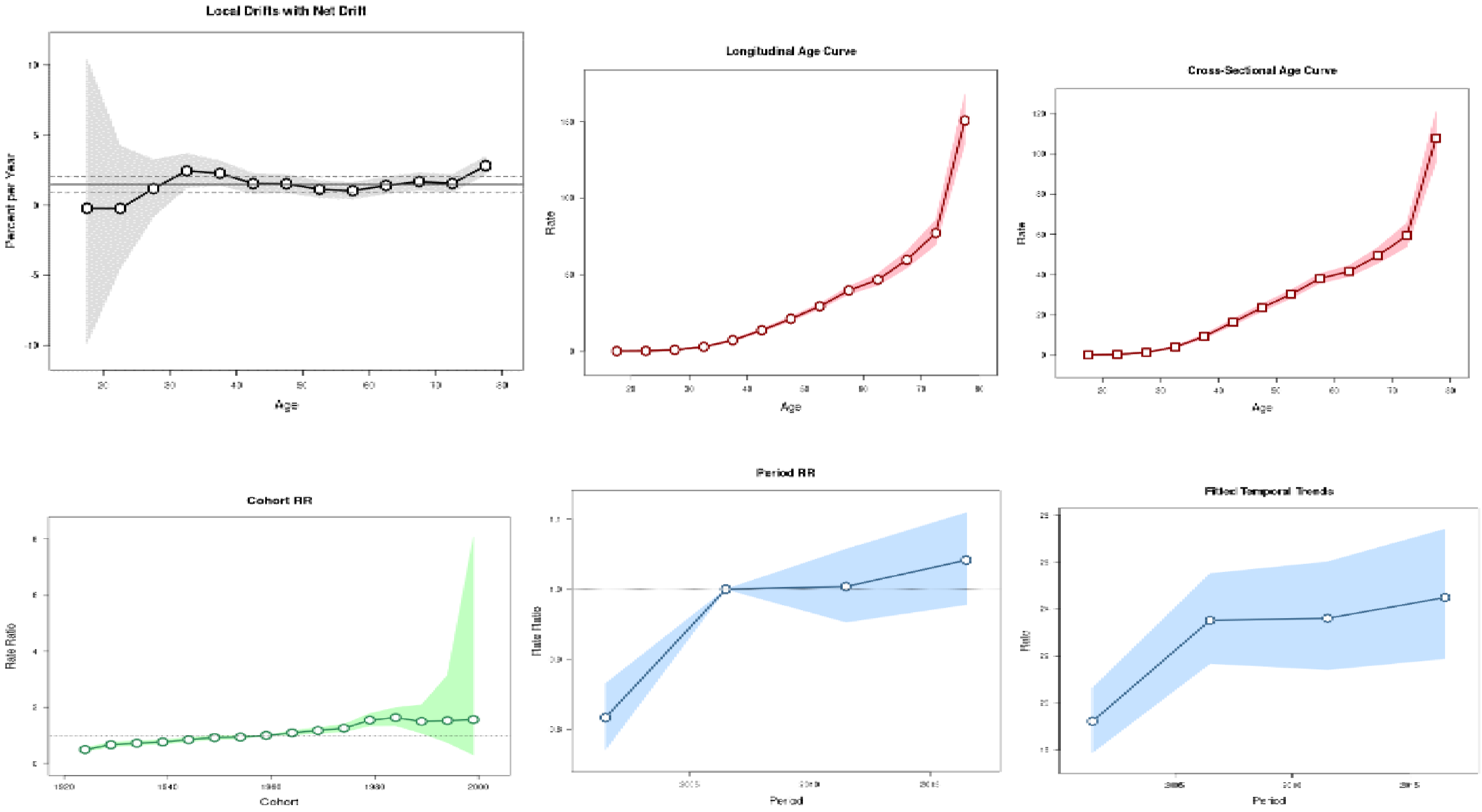

Supplementary Figure 5. Age, period and cohort effects of overall breast cancer mortality in South Africa (1999- 2018)

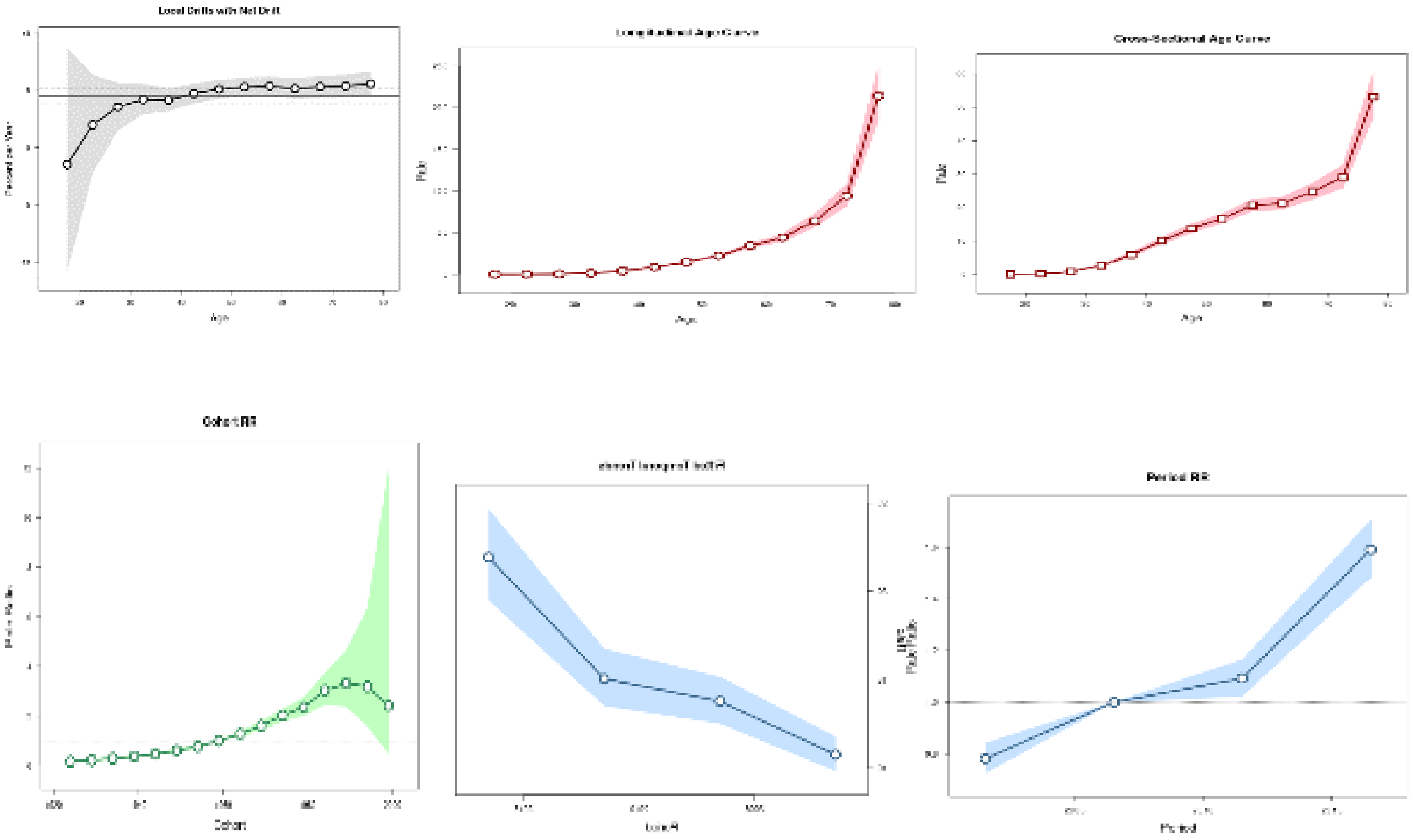

Supplementary Figure 6. Age, period and cohort effects of ethnic breast cancer mortality among Blacks in South Africa (1999- 2018) (Local drift, fitted temporal trends, longitudinal age curve, cross sectional age curve, period effect and cohort effect)

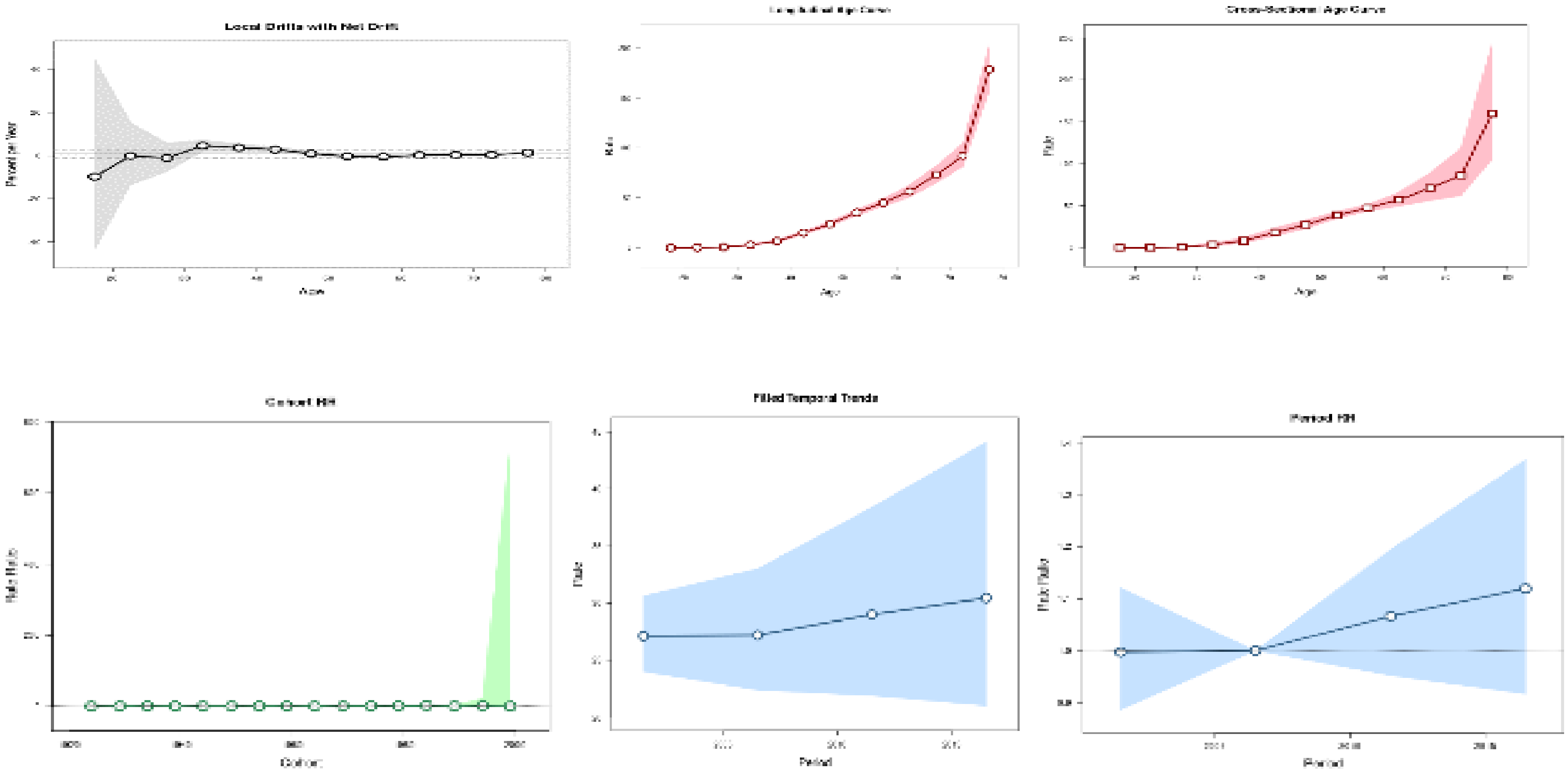

Supplementary Figure 7. Age, period and cohort effects of ethnic breast cancer mortality among Whites in South Africa (1999- 2018). (Local drift, fitted temporal trends, longitudinal age curve, cross sectional age curve, period effect and cohort effect)

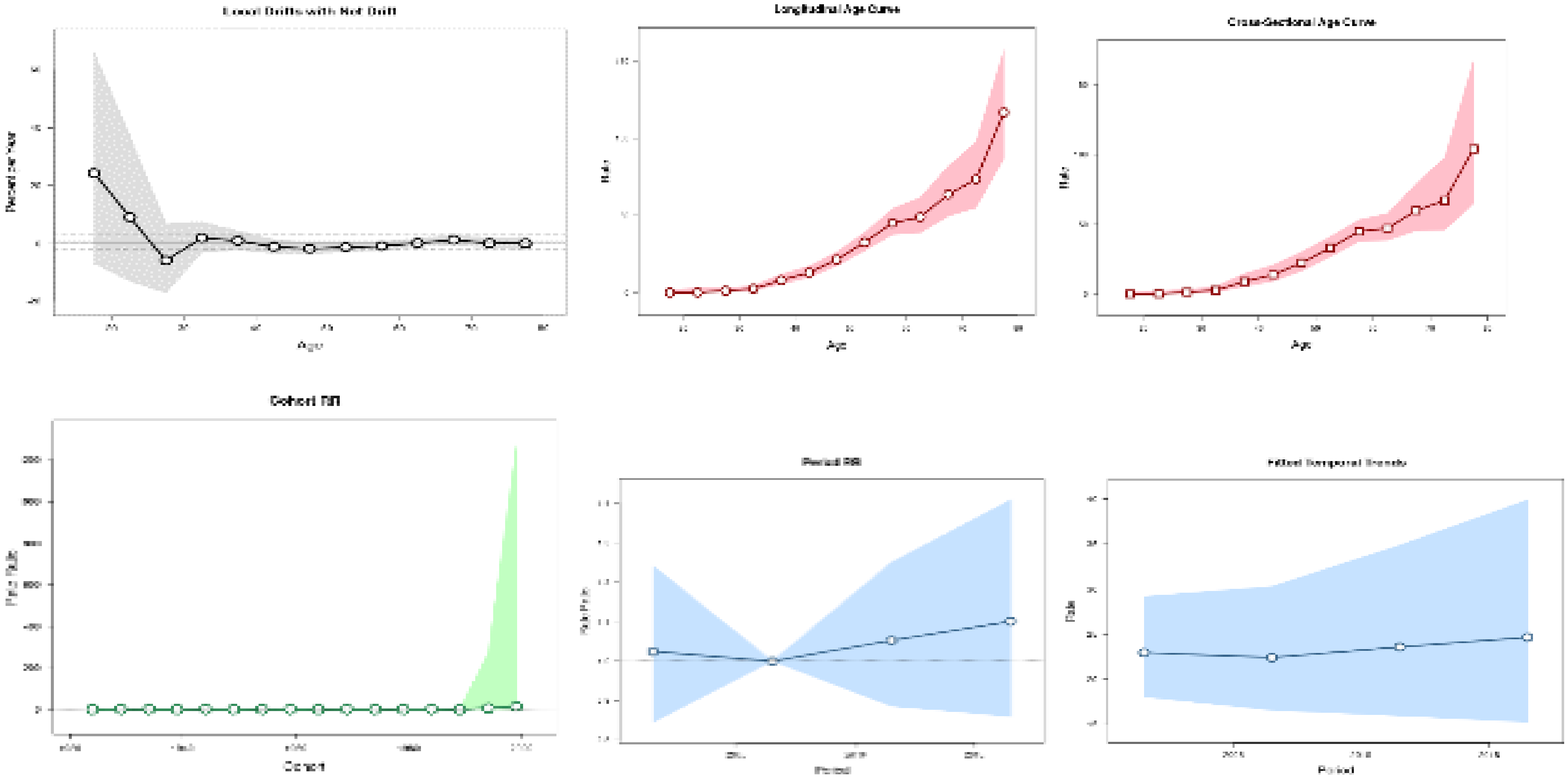

Supplementary Figure 8. Age, period and cohort effects of ethnic breast cancer mortality among Indian/Asian in South Africa (1999- 2018). (Local drift, fitted temporal trends, longitudinal age curve, cross sectional age curve, period effect and cohort effect)

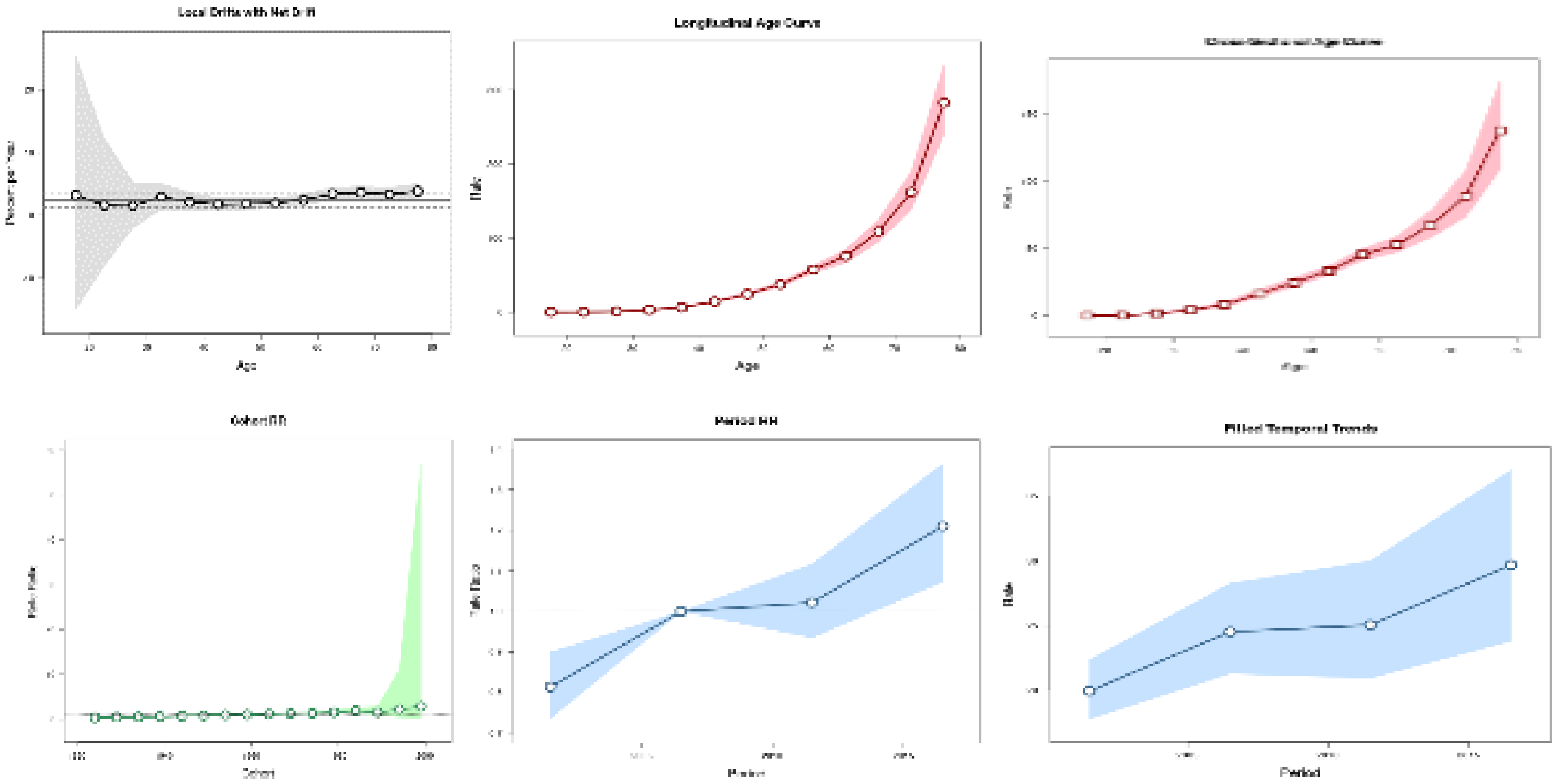

Figure 9. Age, period and cohort effects of ethnic breast cancer mortality among Coloureds in South Africa (1999- 2018)

(Local drift, fitted temporal trends, longitudinal age curve, cross sectional age curve, period effect and cohort effect)
